# Filamin actin-binding and titin-binding fulfill distinct functions in Z-disc cohesion

**DOI:** 10.1371/journal.pgen.1006880

**Published:** 2017-07-21

**Authors:** Nicanor González-Morales, Tristan K. Holenka, Frieder Schöck

**Affiliations:** Department of Biology, McGill University, Montreal, Quebec, Canada; University of Utah, UNITED STATES

## Abstract

Many proteins contribute to the contractile properties of muscles, most notably myosin thick filaments, which are anchored at the M-line, and actin thin filaments, which are anchored at the Z-discs that border each sarcomere. In humans, mutations in the actin-binding protein Filamin-C result in myopathies, but the underlying molecular function is not well understood. Here we show using *Drosophila* indirect flight muscle that the filamin ortholog Cheerio in conjunction with the giant elastic protein titin plays a crucial role in keeping thin filaments stably anchored at the Z-disc. We identify the filamin domains required for interaction with the titin ortholog Sallimus, and we demonstrate a genetic interaction of filamin with titin and actin. Filamin mutants disrupting the actin- or the titin-binding domain display distinct phenotypes, with Z-discs breaking up in parallel or perpendicularly to the myofibril, respectively. Thus, Z-discs require filamin to withstand the strong contractile forces acting on them.

## Author summary

The Z-disc is a macromolecular complex required to attach and stabilize actin thin filaments in the sarcomere, the smallest contractile unit of striated muscles. Mutations in Z-disc-associated proteins typically result in muscle disorders. Dimeric filamin organizes actin filaments, localizes at the Z-disc in vertebrates and causes muscle disorders in humans when mutated. Despite its clinical relevance, the molecular function of filamin in the sarcomere is not well understood. Here we use *Drosophila* muscles and an array of filamin mutations to address the molecular and cell biological function of filamin in the sarcomere. We show that filamin mainly serves as a Z-disc cohesive element, binding both thin filaments and titin. This configuration enables filamin to act as a bridge between thin filaments and the elastic scaffold protein titin from the adjacent sarcomere, maintaining sarcomere stability during muscle contraction.

## Introduction

Arguably, the most complex actin-related cellular structure is the sarcomere, the basic contractile unit of muscle cells. The sarcomere consists of antiparallel actin thin filaments and myosin thick filaments. The thin filaments are anchored to a big protein complex termed the Z-disc at both ends of the sarcomere. In the center of the sarcomere is the M-line, another giant protein complex, that docks the thick filaments. The Z-disc is part of the I-band region, characterized by the absence of myosin. The M-line is at the center of the H-zone region, devoid of actin [1]. The sliding of thick filaments along the thin filaments pulls the Z-disc towards the M-line, representing the basis of muscle contraction [1].

The giant protein titin serves as a molecular spring and provides the passive elasticity of muscles. Titin, which can be as long as 1 μm, extends half a sarcomere and links the myosin thick filaments and the Z-disc [2]. During sarcomere assembly, titin guides thick and thin filament assembly, controls the structure and size of thick filaments and the length of the relaxed sarcomere [2]. Finally, thin filaments from adjacent sarcomeres are crosslinked by α-actinin at the Z-disc creating an array of tandemly arranged sarcomeres [1–3].

Due to the profoundly complex nature of the sarcomere and despite the huge amount of research devoted to it, many aspects of sarcomere assembly have remained elusive. Notably, many sarcomeric proteins are associated with human myopathies and despite their clinical relevance the exact function that many of these proteins play in the sarcomere is not clear.

Filamin was the first actin filament crosslinking protein identified in nonmuscle cells [4]. Filamins are large homodimers that associate at their carboxy termini through a conserved hydrophobic pocket [5]. Each filamin consists of an N-terminal actin-binding domain (ABD) followed by 22–24 immunoglobulin-like (Ig) repeats, the last of which is the dimerization domain [6–8]. The Ig repeats are further subdivided into an extended rod 1 domain, and a more globular rod 2 domain, which can unfold in response to mechanical force and contains most of the binding sites for around 90 binding partners identified to date [6–12].

Vertebrates have three filamin proteins, FLNa, FLNb, and FLNc. FLNa and b are widely and similarly expressed throughout development, whereas FLNc is restricted largely to cardiac and skeletal muscles [8]. Mutations in filamins result in a wide variety of congenital anomalies, but due to its expression, only mutations in FLNc result in muscle disorders, including muscular dystrophies, myofibrillar myopathy, distal myopathy and cardiomyopathy [13]. FLNc localizes to Z-discs and FLNc-deficient mice exhibit reduced muscle mass and structural defects, like loss of distinct Z-disc components [14–16]. Despite the clinical relevance, the exact function of FLNc in muscles has remained elusive.

The *Drosophila* gene encoding filamin was first identified because of its critical role in the assembly of ovarian ring canals, and was therefore called *cheerio* (*cher*) [17–19]. *Drosophila* filamin is highly conserved with vertebrate filamins lacking only two Ig repeats in the rod 1 domain [10]. Cheerio recruits actin filaments to the ring canal and likely tethers the ring canal to the plasma membrane [18–20]. Cher also functions as part of a perinuclear actin meshwork that connects actin cables to the nuclei, ensuring proper localization [21]. Like FLNa and FLNb, Cheerio plays important roles in enhancing tumor malignancy [22]. In the nervous system, it is required for proper peripheral motor axon guidance, in memory formation, and at the neuromuscular junction as a signaling hub [23–25]. Cheerio was also uncovered in a genome-wide screen for genes required in muscles [26], but has not been further analyzed in muscles except as an interaction partner of small heat shock protein CryAB [27].

Here we investigate filamin function in muscles, through detailed phenotypic analysis of Cher in the indirect flight muscles (IFM). We focused on the IFM because of their structural similarities to vertebrate skeletal muscles and because they have the most structurally stereotyped sarcomeres, allowing the detection of subtle defects [28].

We show that loss of filamin results in distinct sarcomere phenotypes. Mainly, we observe the detachment of actin thin filaments from the Z-disc both perpendicular and parallel to the sarcomere axis. We show that filamin actin-binding is required for keeping thin filaments anchored to the Z-disc, while filamin binding to the titin homolog Sallimus (Sls) is required for stabilizing the position of thin filaments perpendicular to the myofibril axis. Our data provide an explanation for the function of filamin in muscle and a framework for understanding some human FLNc myopathies [29].

## Results

### Cher isoforms are components of the Z-disc

**Fig 1:**
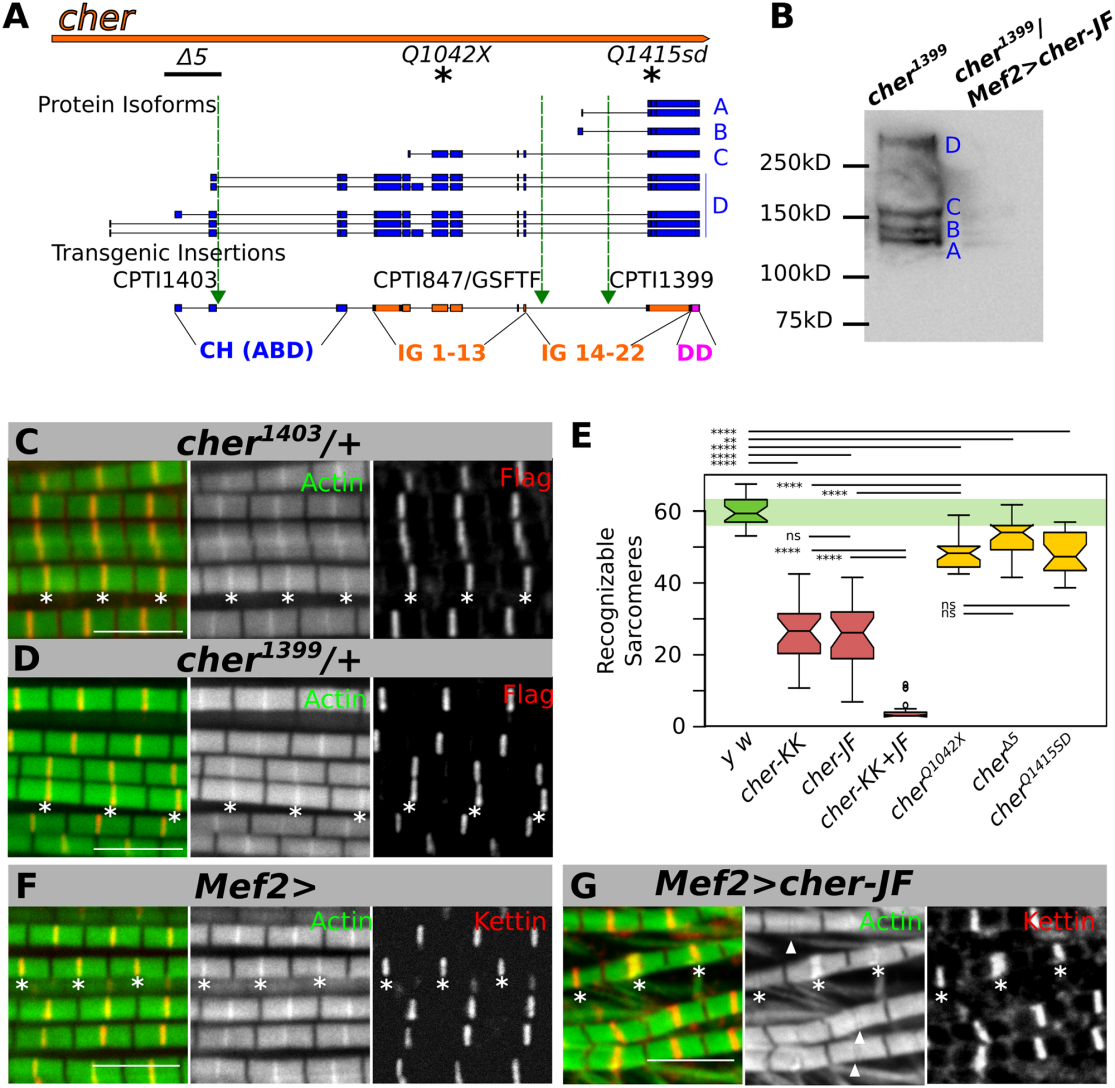
Cher localizes to the Z-disc in the IFM and is necessary for sarcomere structural stability. **(A**) *cher* genomic region showing several transcripts and several protein isoforms that can be grouped based on their molecular weight into 4 groups (CherA-D). Green triangles represent transgenic insertion sites of protein-trap lines, each bearing a splice acceptor site followed by a Venus and a Flag tag. The protein domains encoded by different exons are indicated at the bottom: CH (ABD), calponin homology domain 1 and 2 comprises the actin-binding domain; Ig, immunoglobulin-like domains; DD, dimerization domain. Asterisks denote location of the premature stop point mutants in *cher*^*Q1042x*^ and *cher*^*Q1415sd*^. The *cher*^*Δ5*^ deleted region is denoted by a black line (**B**) Immunoblot from *cher*^*CPTI1399*^ thoraces incubated with anti-Flag antibody reveals 4 Cher isoforms, specifically depleted in *Mef2-Gal4*>UAS-*cher-JF* RNAi. (**C, D**) IFM confocal images from heterozygous Flag-tagged Cher traps stained with anti-Flag antibody to visualize the tagged isoforms. In all images, asterisks mark Z-discs of a selected myofibril immediately above the asterisks. (**C**) All Cher isoforms, revealed by the *cher*^*CPTI1403*^ protein trap, localize at the Z-disc. (**D**) Long Cher isoforms tagged by *cher*^*CPTI1399*^ localize to the Z-disc. (**E**) Quantification of recognizable sarcomeres in control, Cher-depleted, and *cher* mutant IFM. Statistical significance assessed by one-way ANOVA with post hoc Tukey: n.s. = not significant, ** = P ≤ 0.01, **** = P ≤ 0.0001. (**F**, **G**) Confocal images from control and Cher-depleted IFM, stained with anti-Kettin antibody to visualize Z-discs in red and phalloidin to visualize actin thin filaments in green. (**F**) Regular sarcomeric structure in *Mef2-Gal4* control flies, (**G**) Depletion of Cher in *Mef2-Gal4*, *UAS-cher-JF* flies results in severe sarcomeric disorganization. Arrowheads indicate reduced actin staining at the Z-disc. Scale bars: 5 μm.

The *cher* gene spans a 34 kb region and produces at least 10 different transcript isoforms whose encoded proteins can be divided according to FlyBase into 4 molecular size groups: the small isoforms, here referred to as CherA and B (90–100 kD); a medium-sized isoform, CherC (150 kD), and CherD, containing all the big isoforms (240–260 kD) (Fig 1A) [30].

To assess the localization of Cher in the IFM we used 4 different protein trap lines that introduce a 30 kD Venus Flag-tagged artificial exon directly into the *cher* gene [31,32]. Due to their specific location inside the *cher* gene, *cher*^*CPTI1399*^ tags all protein isoforms, *cher*^*CPTI847*^ and *cher*^*GFSTF*^ tag CherC and CherD isoforms; while *cher*^*CPTI1403*^ tags only CherD isoforms (Fig 1A and S1D Fig). *cher*^*CPTI847*^ and *cher*^*CPTI1403*^ proteins are not fully functional, because homozygotes cause female infertility. *cher*^*CPTI1399*^ and *cher*^*GFSTF*^ are homozygously fertile.

We first evaluated by immunoblotting the presence of Cher isoforms in the adult thorax using *cher*^*CPTI1399*^, because this line should tag most predicted isoforms of *cher*. All Cher isoform groups were detected using this assay (Fig 1B). To test the specificity of the detected bands we knocked down *cher* expression specifically in muscles using an RNAi directed against all isoforms under the control of the *Mef2* expression pattern using the Gal4-UAS system: *Mef2*>*cher-JF* RNAi. Consistently, Flag-positive bands were no longer detected indicating that all bands correspond to Cher isoforms (Fig 1B). We then analyzed the localization of these protein traps in the IFM using heterozygotes. While direct Venus fluorescence was barely detectable, anti-Flag staining was detected at the Z-disc, colocalizing with the peak of the actin signal (Fig 1C and 1D, S1A Fig). We also used line scans to better assess their localization; all Cher-GFP trap lines show identical localization profiles peaking at the Z-disc, indicating that all filamin isoforms colocalize at the Z-disc (S1C Fig).

All three Cher protein traps localize to the Z-disc, suggesting that the small CherA isoforms contain the Z-disc localization information. To further test this, we expressed the smallest CherA isoform fused to GFP in IFM. Consistently, CherA-GFP also localizes to the Z-disc (S1B Fig). Thus, all Cher isoforms are components of the Z-disc and CherA, containing the last 8 Ig-like domains is sufficient for Z-disc localization.

### Cher depletion results in sarcomeric defects

There are three available RNAi lines directed against all cher isoforms. We first assessed the efficiency of these lines by testing their ability to render flies flightless. Two of these lines, *cher*^*JF02077*^ and *cher*^*KK107451*^, in combination with the muscle-specific driver *Mef2-Gal4* led to a completely penetrant flightless phenotype. We then evaluated the IFM sarcomeric defects underlying the flying defects by first quantifying sarcomere numbers visible in confocal images. The number of recognizable sarcomeres is reduced by half in individual RNAi lines *cher*^*KK107451*^ and *cher*^*JF02077*^, and is even further reduced in the double knock-down (Fig 1E and S1E and S1F Fig), indicating strong defects in myofibril assembly or maintenance. A similar yet less severe sarcomere reduction phenotype is observed in other *cher* mutants (Fig 1E). We proceeded to analyze the sarcomeres of *cher*^*JF02077*^ flies in more detail.

In control flies, individual IFM sarcomeres are highly stereotyped regular structures with a perfectly defined Z-disc, revealed by the highest intensity peak of actin staining colocalizing with the Z-disc marker Kettin (Fig 1F). In *Mef2>cher*^*JF02077*^ flies the stereotypical sarcomere pattern is often lost, resulting in shredded myofibrils. We also observed almost normal sarcomeres, but with less actin at the Z-disc, which we call a widened Z-disc phenotype (arrowheads in Fig 1G). Infrequently, sarcomeres also exhibit very high actin accumulation at the Z-disc (middle asterisk in Fig 1G). Similar defects were seen in *Mef2>cher*^*KK107451*^ flies, confirming the specificity of the phenotype (S1E Fig). When both RNAi lines are combined, *Mef2>cher*^*JF02077*^ *cher*^*KK107451*^ flies display a complete loss of sarcomeric structure, where actin staining is diffuse and continuous and Kettin staining is lost (S1F Fig).

**Fig 2:**
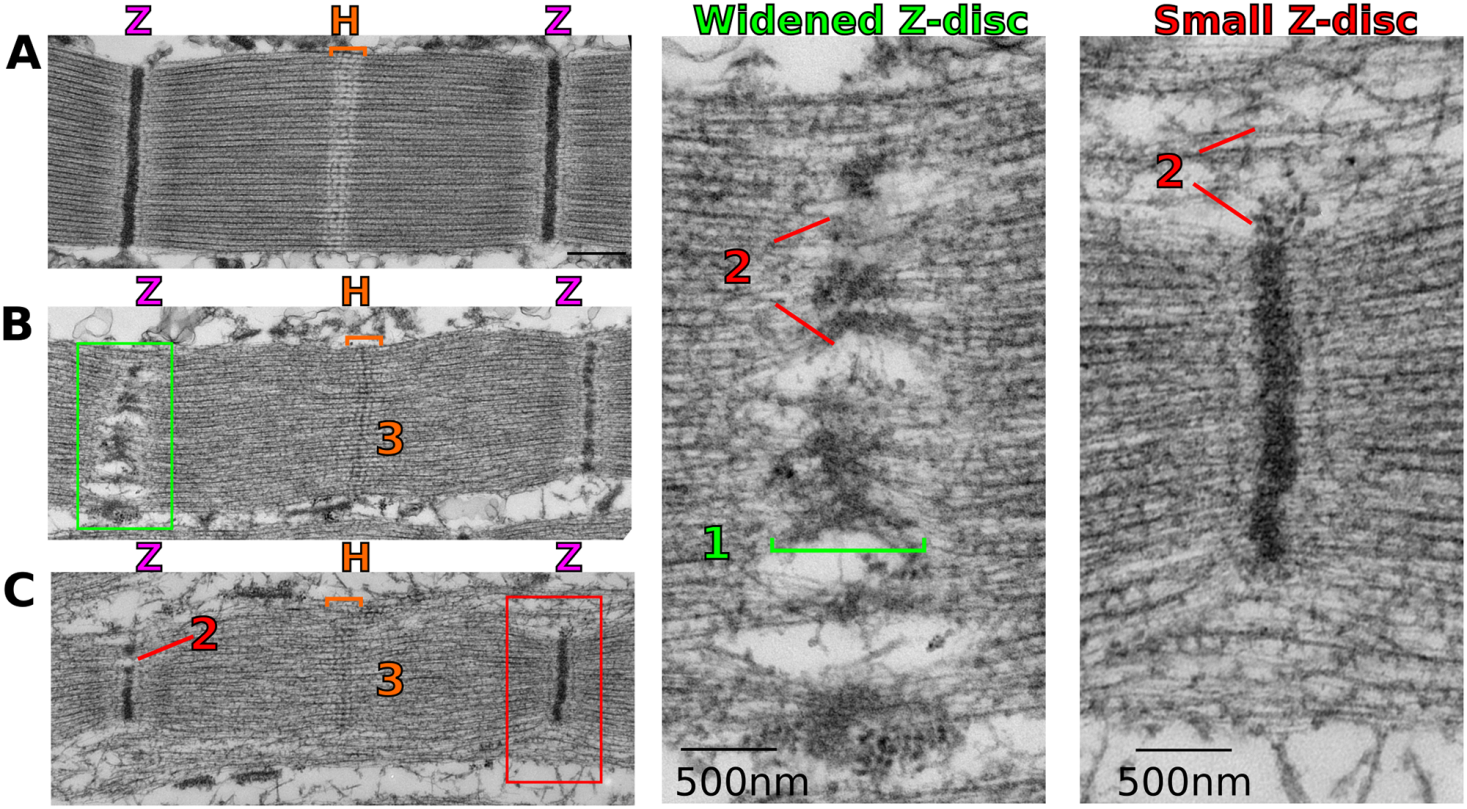
Distinct sarcomere phenotypes revealed by electron microscopy. (**A**) TEM image of a wild type sarcomere. Z stands for the Z-disc; H stands for the H-zone and is indicated by orange brackets; (**B**, **C**) *Mef2-Gal4*, *UAS-cher-JF* sarcomeres show widened Z-discs (green box, shown enlarged on the right), smaller than normal Z-discs (red box, shown enlarged on the right) and actin accumulation at the H-zone (orange brackets). Light green bracket denotes the width of the Z-disc. Numbers correspond to (1) a widened Z-disc, (2) a smaller or fractured Z-disc, and (3) actin incorporation into the H-zone. Scale bars: 500 nm.

To better analyze the IFM phenotype upon Cher depletion, we analyzed the sarcomere phenotype at a higher resolution using transmission electron microscopy (TEM). Control sarcomeres show an electron-dense structure corresponding to the Z-disc with a lighter surrounding area, together corresponding to the I-band. The highly ordered parallel actin thin filaments are readily seen, except in the center of the sarcomere, the H-zone, where actin is excluded (Fig 2A). In *Mef2>cher*^*JF02077*^ sarcomeres three distinct phenotypes were observed: 1) a widened Z-disc, 2) a smaller or fractured Z-disc, and 3) actin incorporation into the H-zone (numbers in Fig 2).

### CherD stably attaches actin thin filaments at the Z-disc

**Fig 3:**
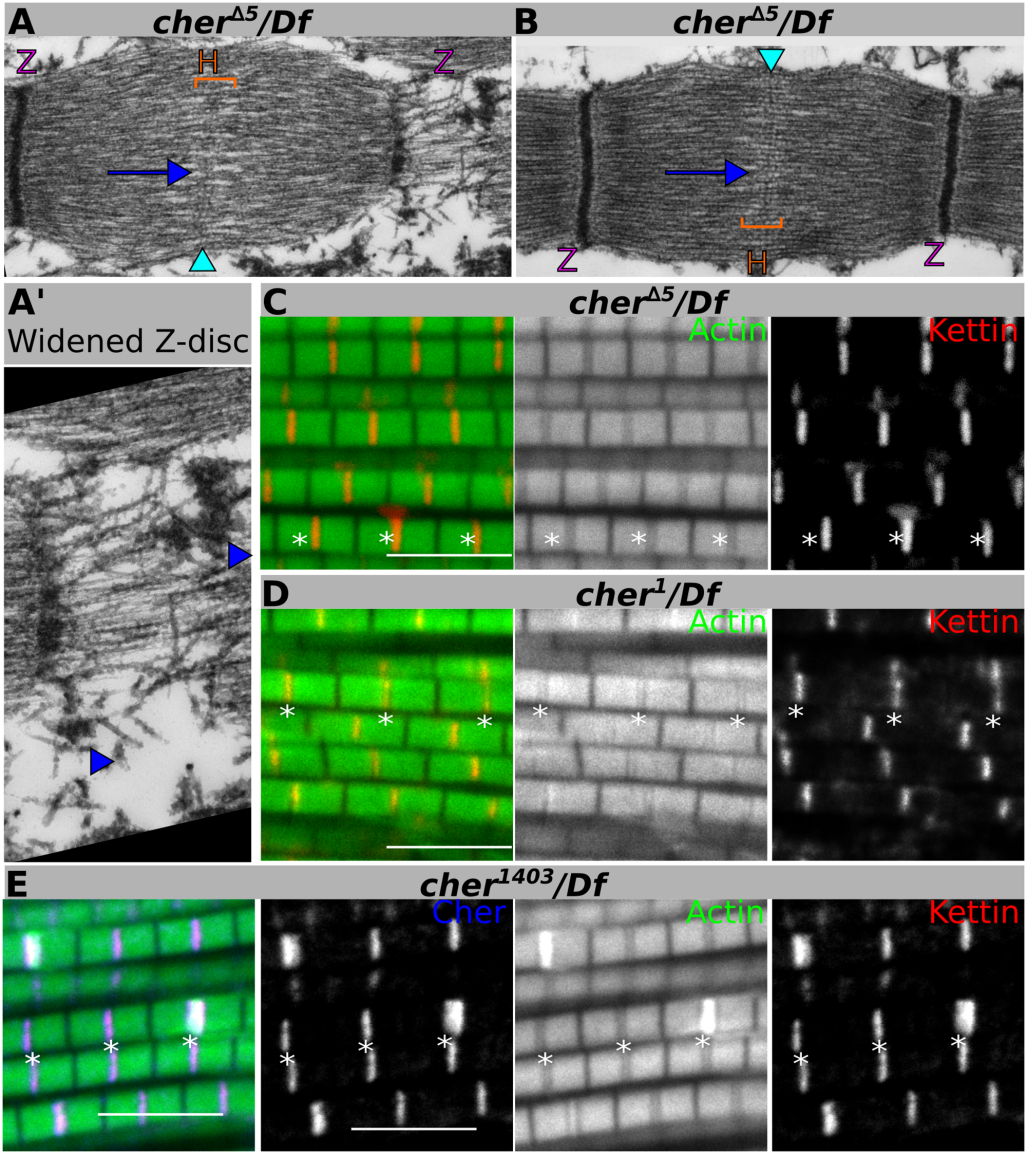
Disruption of Cher-actin binding accounts for the widened Z-disc phenotype. (**A**, **B**) Electron micrographs of *cher*^*Δ5*^ mutant sarcomeres show Z-discs ripped apart (enlarged in **A’**, blue arrowheads indicate the left and right borders of a ripped-apart Z-disc) and actin accumulation at the H-zone (blue arrows), without affecting the M-line (light blue arrowheads). (**C**-**E**) Confocal images of IFM stained with phalloidin to visualize actin thin filaments in green, anti-Kettin antibody to visualize Z-discs in red, and anti-Flag antibody to visualize Cher isoforms in blue. (**C**, **D**) Both *cher*^*Δ5*^ and *cher*^*1*^ mutants show widened Z-discs with reduced actin staining in many sarcomeres. (**E**) The *cher*^*1403*^ actin-binding mutant also phenocopies the widened Z-disc phenotype. Scale bars: 5 μm.

The CherD isoforms correspond to the big Cher isoforms, the only isoforms containing the CH actin-binding domain (ABD). Given that Cher depletion results in thin filament disorganization mostly at the Z-disc, we hypothesized that CherD, through the ABD, might serve as a secondary attachment of thin filaments to the Z-disc. To test this, we used the *cher*^*Δ5*^ mutant bearing a 2.4 kb deletion uncovering the CherD transcription start site thus reducing CherD protein levels while leaving the other isoforms intact [20,24] (Fig 1A and S2A Fig). TEM of *cher*^*Δ5*^ mutant sarcomeres revealed three phenotypes: 1) a widened Z-disc resulting from a splitting or opening of the Z-disc (Fig 3A and 3A’), 2) actin accumulation at the H-zone (blue arrow in Fig 3A and 3B) and 3) a smaller or fractured Z-disc (Fig 3A and 3A’). These phenotypes are similar to the spectrum of phenotypes observed in *Mef2>cher*^*JF02077*^ sarcomeres, but of lower penetrance (see Fig 1E), suggesting that C-terminal filamin isoforms can rescue some aspects of filamin function at the Z-disc.

We then used confocal microscopy to analyze other *cher* mutants that disrupt the CherD isoforms. First, we evaluated *cher*^*Δ5*^ mutant IFM stained for Kettin and actin. In contrast to a control staining (Fig 1F), *cher*^*Δ5*^ IFM display a widened Z-disc phenotype, evident by the lack of actin staining (Fig 3C). Actin accumulation at the H-zone was also occasionally observed by confocal microscopy (S3B Fig, compare to S3A Fig). Both phenotypes agree with our TEM data. As the widened Z-disc phenotype was easier to distinguish by confocal microscopy we used it as a readout for analyzing other *cher* mutants.

We first tested one of the original *cher* alleles to be isolated, *cher*^*1*^, that has been shown to greatly reduce CherD protein levels without affecting CherA/B [18,25]. Confocal microscopy images of *cher*^*1*^ mutant IFM stained for Kettin and actin confirmed the widened Z-disc phenotype, suggesting that the large CherD isoforms account for this phenotype (Fig 3D).

CherD isoforms are the only isoforms containing an actin-binding domain begging the question of whether actin binding alone is responsible for the widened Z-disc phenotype. To test this hypothesis, we used the *cher*^*CPTI1403*^ protein trap that inserts a Venus-Flag tag into the first CH domain, leaving a Cher protein with severely compromised actin binding [22,31] (S2D Fig, compare to S2B and S2C Fig). In *cher*^*CPTI1403*^ mutants widened Z-discs were evident, together with a widening of Kettin staining and loss of actin staining. CherD^*CPTI1403*^ staining at the Z-disc also appeared wider than normal (Fig 3E). As for *Mef2>cher*^*JF02077*^, sarcomeres with very high actin accumulations at the Z-disc were also observed occasionally (Fig 3E). These data together support the notion that the ABD only found in CherD isoforms is required for keeping actin thin filaments stably attached at the Z-disc.

### Removal of one copy of *Actin88F* enhances Cher phenotype

The *Actin88F* gene produces the main actin protein in the IFM and its expression is also largely restricted to the IFM. Due to these two characteristics, *Act88F* homozygous null mutants result in viable flightless flies with complete absence of actin in the IFM [33,34]. *Act88F* null heterozygous mutants display a sensitive dominant flightless phenotype suitable for assessing genetic interactions.

**Fig 4:**
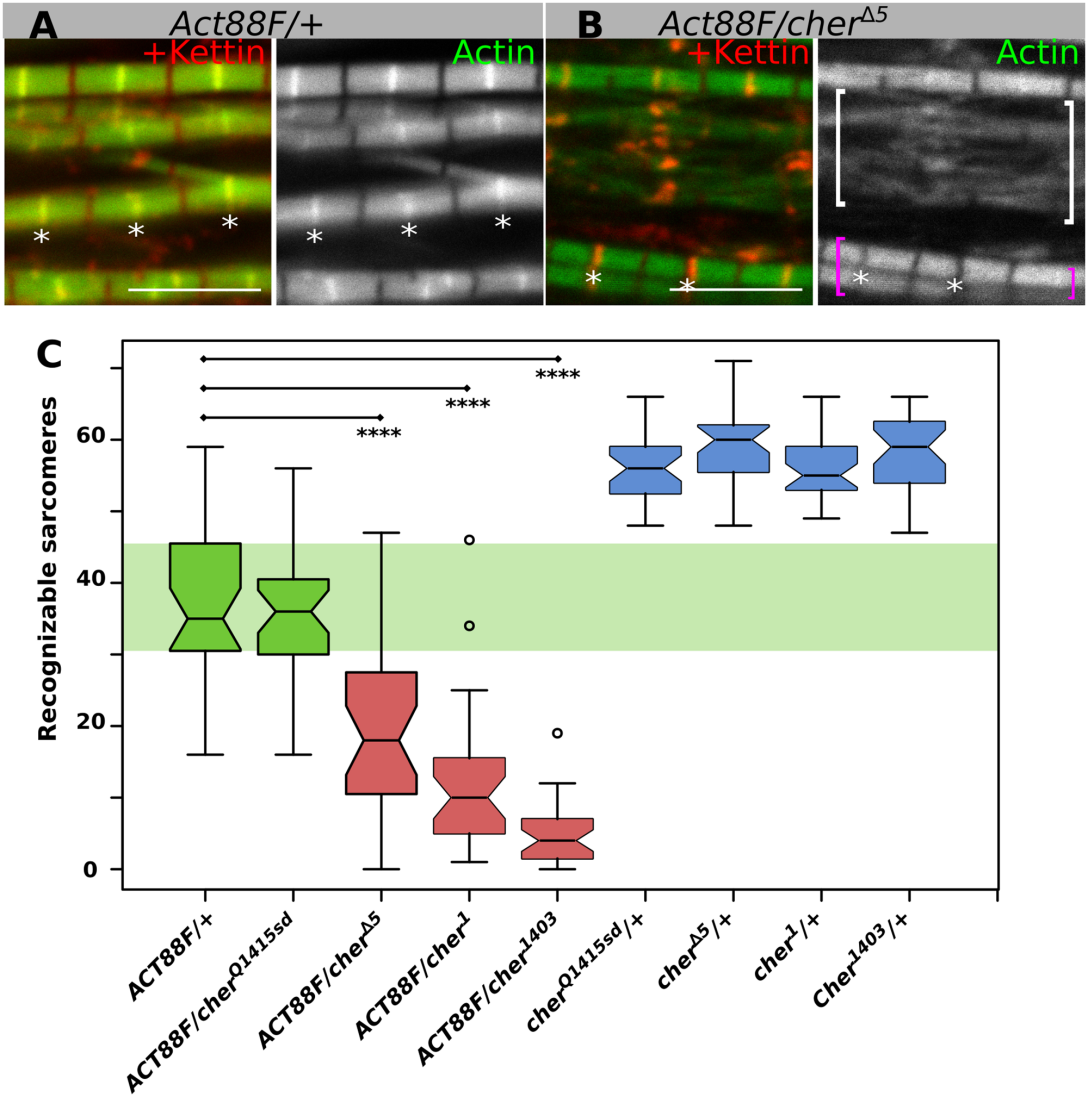
Genetic interaction between *Act88F* and *cher*. (**A**, **B**) Confocal images of IFM stained with phalloidin to visualize actin thin filaments in green and anti-Kettin antibody to visualize Z-discs in red. (**A**) *Act88F* heterozygotes have a moderate sarcomere phenotype. (**B**) Transheterozygous *Act88F*/*cher*^*Δ5*^ sarcomeres display Kettin aggregates and stronger phenotypes, including widened Z-discs. White brackets denote area of completely frayed and thus uncountable sarcomeres, purple brackets show recognizable sarcomeres. Scale bars: 5 μm. (**C**) Quantification of recognizable sarcomeres in *Act88F* heterozygotes or in transheterozygous *Act88F cher* mutants. Only mutants affecting the actin-binding CherD isoform genetically interact with *Act88F*. Statistical significance assessed by one-way ANOVA with post hoc Tukey: **** = P ≤ 0.0001.

In *Act88F*^*KM88*^ heterozygotes IFM sarcomeres appear relatively normal except for the occasional splitting of myofibrils (Fig 4A). In contrast, in *Act88F*^*KM88*^ *cher*^*Δ5*^ transheterozygotes sarcomeres are damaged; frayed sarcomeres and widened I-bands are regularly observed (Fig 4B). To quantify the genetic interaction between *Act88F* and *cher*, we counted the number of recognizable sarcomeres compared to areas where myofibrils are too frayed to distinguish individual sarcomeres (Fig 4C). Consistently, *cher*^*Δ5*^, *cher*^*1*^ and *cher*^*1403*^ enhance the *Act88F* dominant phenotype, that is, reduce the number of recognizable sarcomeres, whereas *cher*^*Q1415sd*^, a C-terminal filamin mutant, does not genetically interact with *Act88F*. The specific genetic interaction of the *cher* ABD mutants with actin further confirms the role of the filamin ABD in tethering actin thin filaments to the Z-disc.

### C-terminal Cher mutants reveal distinct sarcomere phenotypes

The depletion of all Cher isoforms leads to severe sarcomere disintegration (Fig 1E). However, *cher*^*1*^ and *cher*^*Δ5*^ mutants which completely abolish the CherD isoform, only show milder sarcomere defects, suggesting that the remaining isoforms can rescue some aspects of filamin function. To test this hypothesis, we analyzed two mutants that introduce early stop codons at molecularly defined positions: *cher*^*Q1415sd*^ introduces a premature stop codon after Ig 15 leaving the last 7 Ig domains untranslated and *cher*^*Q1042x*^ introduces a stop codon after Ig 11. Due to the complexity of *cher* alternative splicing and internal transcription start sites, *cher*^*Q1042x*^ only affects the CherD and C isoforms while *cher*^*Q1415sd*^ affects all isoforms (Fig 1A and S2A Fig).

**Fig 5:**
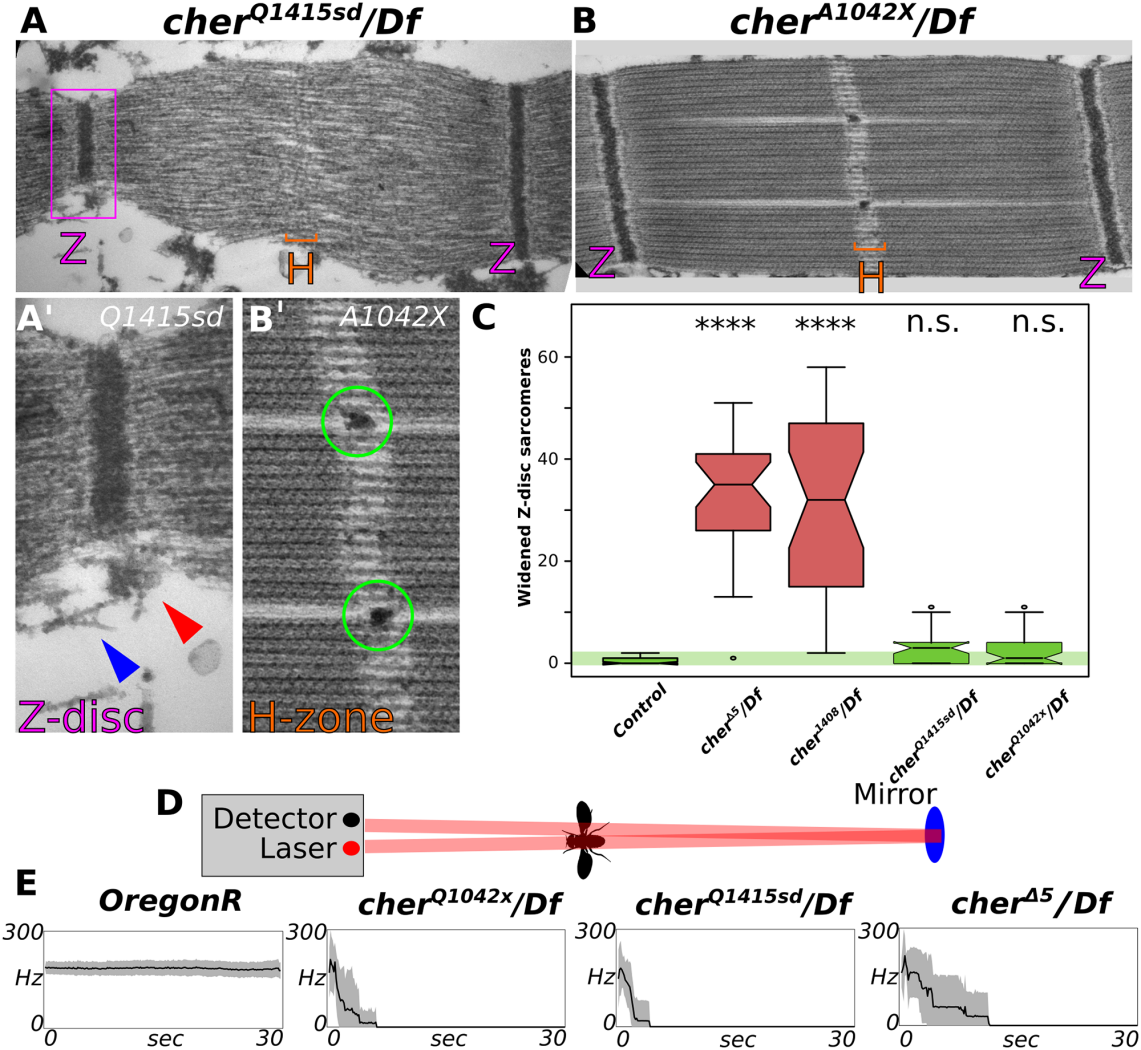
C-terminal Ig domains maintain Z-disc cohesion perpendicular to the myofibril. (**A**, **B**) TEM of two *cher* C-terminal mutants. (**A**) *cher*^*Q1415sd*^ mutant sarcomere shows actin accumulation at the H-zone and a smaller Z-disc (purple box, enlarged in **A’**). (**A’**) Z-disc material (red arrowhead) and actin filaments (blue arrowhead) are observed detaching from the myofibril. (**B**) *cher*^*Q1042x*^ mutant sarcomere displays mild defects, with Z-disc material pulled into the H-zone (enlarged in **B’**). (**C**) Quantification of the number of widened Z-discs in different *cher* mutants. Only mutants affecting the actin-binding CherD isoform display a widened Z-disc in the IFM. Statistical significance assessed by one-way ANOVA with post hoc Tukey: n.s. = not significant, **** = P ≤ 0.0001. (**D**) Schematic representation of the laser tachometer used to measure wing beat frequency. (**E**) All *cher* homozygous mutants have very short flying times, compared to the wild type control OregonR. The average of 10 flies with standard deviation is shown.

TEM images of *cher*^*Q1415sd*^ revealed 2 phenotypes in common with *Mef2>cher*^*JF02077*^: 1) a smaller or fractured Z-disc, and 2) actin invasion of the H-zone (Fig 5A and 5A’). TEM images of *cher*^*Q1042x*^ revealed a single phenotype: Z-disc fragments located at the H-zone (Fig 5B and 5B’). The widened Z-disc phenotype was not observed in any of these mutants, suggesting that the N-terminal region before the premature stop codon in *cher*^*Q1415sd*^ is able to rescue the widened Z-disc phenotype. To confirm this result we quantified the number of widened Z-disc sarcomeres in different *cher* mutants using confocal microscopy. Indeed, widened Z-discs were observed with statistical significance only in the ABD mutants *cher*^*Δ5*^ and *cher*^*1403*^, but not in *cher*^*Q1042x*^ or *cher*^*Q1415sd*^ (Fig 5C). Thus, ABD and C-terminal filamin mutants have distinct phenotypes.

### All Cher isoforms are required for sustained flight

We noticed that *cher* mutants were not entirely flightless, in contrast to *Mef2>cher*^*JF02077*^ knock-down flies. Consistent with our previous data, this observation argues that some remaining Cher function is present in our *cher* mutants. To quantify the flight ability of *cher* mutants we used an infrared tachometer to monitor the wing beat frequency of tethered flies (Fig 5D). While control animals can fly at a constant speed, around 200 Hz for several minutes without interruption, *cher* mutants are not able to sustain flight for more than a couple of seconds (Fig 5E), demonstrating the physiological relevance of all mutants.

### Cher and Sls interact in Z-disc cohesion

Resting elasticity of the sarcomere in striated muscle is determined by the giant modular protein titin. In vertebrates, titin reaches from the Z-disc across half the sarcomere up to the M-line. In invertebrates, the function of titin is split between Sls and Projectin. While Projectin associates with the thick filaments in the A-band, Sls spans from the Z-disc to the edge of the A-band [35–37]. Sls isoforms vary highly in size, from 2000 kD to 350 kD, the smallest being Zormin. The main isoform in the IFM is the 500 kD isoform called Kettin [36].

We previously noticed that Kettin staining is affected in some Cher-depleted sarcomeres as well as in *Act88F cher*^*Δ5*^ transheterozygotes (Figs 1G, 3C–3E and 4B). These results suggest that Kettin may be involved in Cher function at the IFM.

**Fig 6:**
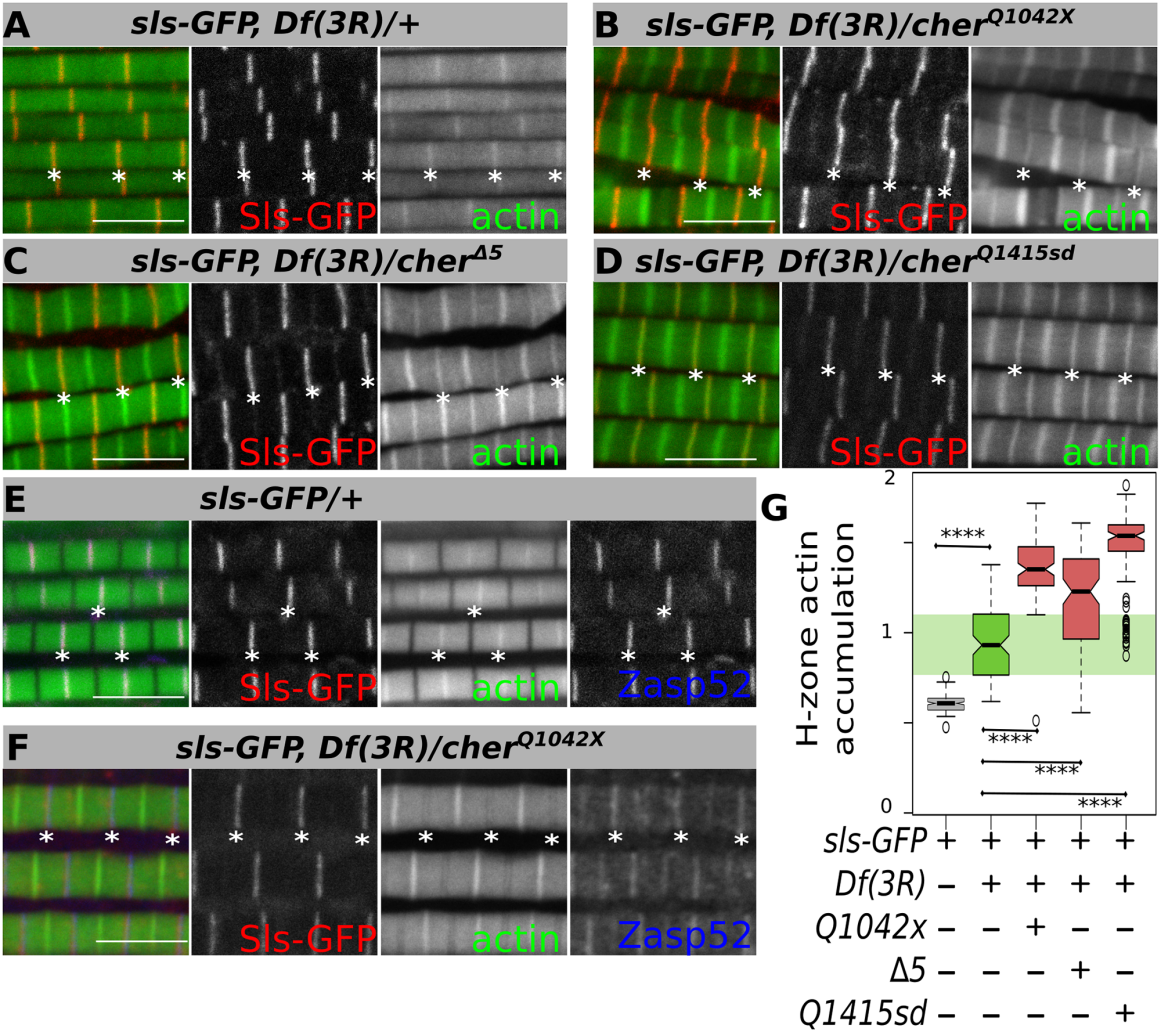
Actin accumulation in the H-zone in different *cher* mutants. (**A**-**D**) Confocal images of IFM stained with phalloidin to visualize actin thin filaments and GFP fluorescence marking Sls at the Z-disc. (**A**) Transheterozygous *sls-GFP Df(3R)Exel6176* mildly accumulate actin at the H-zone. (**B**-**D**) Sarcomeres from *sls-GFP Df(3R)Exel6176* in combination with different *cher* mutants result in actin aggregates at the H-zone, while Sls-GFP remains at the Z-disc. (**E**, **F**) Confocal images of IFM stained with phalloidin to visualize actin thin filaments, anti-Zasp52 to visualize Z-disks, and GFP fluorescence marking Sls at the Z-disc. (**E**) *sls-GFP* heterozygotes show GFP at the Z-disc together with Zasp52. (**F**) In *sls-GFP Df(3R)Exel6176/cher*^*Q1042x*^ sarcomeres Sls-GFP and Zasp52 still colocalize at the Z-disc. (**G**) Quantification of H-zone actin accumulation in different *cher* mutant backgrounds shown as box plots. Statistical significance assessed by one-way ANOVA with post hoc Tukey: **** = P ≤ 0.0001. Scale bars: 5 μm.

We first analyzed *sls*^*ZCL2144*^, which bears a GFP exon trap, predominantly labels Kettin in IFM and localizes to the Z-disc. It has no muscle phenotype heterozygously (Fig 6E), but shows IFM defects homozygously [38]. We first recombined *sls*^*ZCL2144*^ with *Df(3R)Exel6176*, a deficiency uncovering the *cher* locus. This transheterozygous combination led to mild sarcomere defects consisting of actin accumulation at the H-zone, evident by the lack of separation of individual sarcomeres in actin stainings (Fig 6A and 6G). These actin-related phenotypes suggested that Cher together with Sls/Kettin might be involved in keeping actin anchored at the Z-disc. To further test this, we analyzed the homozygous mutant conditions of our *cher* mutants. Without the addition of *sls*^*ZCL2144*^, H-zone actin accumulation is not visible by confocal microscopy in *cher*^*Q1415sd*^ or *cher*^*Q1042x*^ mutants and present only at a low frequency in *cher*^*Δ5*^ mutants (S3 Fig). Interestingly, *cher*^*Q1042x*^ in combination with *sls*^*ZCL2144*^ resulted in a highly significant enrichment of actin at the H-zone (Fig 6B and 6G). *cher*^*Δ5*^ and *cher*^*Q1415sd*^ in combination with *sls*^*ZCL2144*^ also led to strong H-zone actin accumulation (Fig 6C, 6D and 6G). In many sarcomeres, the H-zone now looks like the Z-disc, if only the actin staining is considered. We therefore confirmed the location of the Z-disc by using Zasp52 as a second Z-disc marker, showing that Sls/Kettin remains at the Z-disc (Fig 6F). Our data indicate that Sls/Kettin genetically interacts with Cher and together with Cher mediates actin anchorage to the Z-disc.

**Fig 7:**
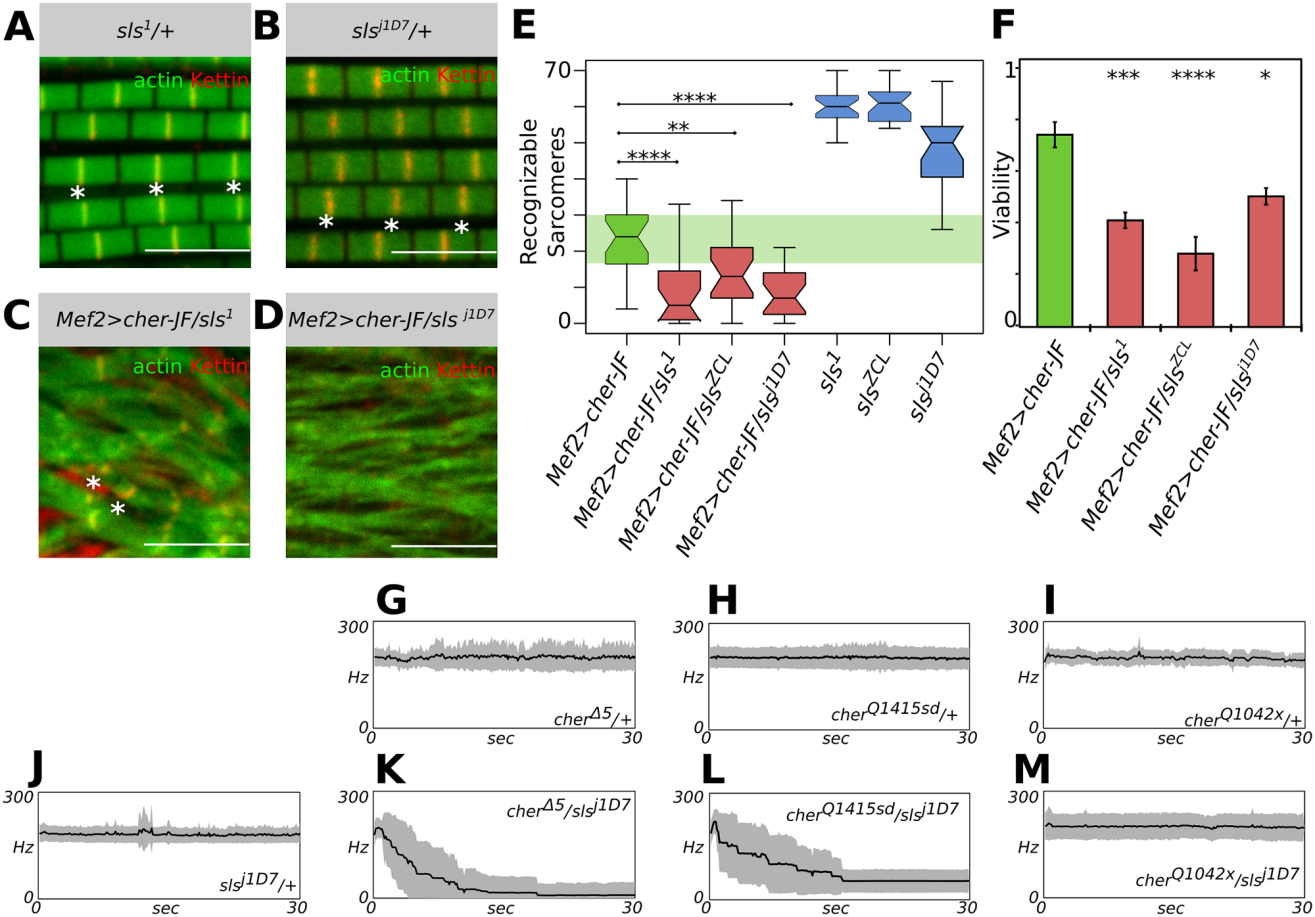
Cher interaction with Sls is essential for maintaining sarcomere structure. (**A**-**D**) Confocal images of IFM stained with phalloidin to visualize actin thin filaments and anti-Kettin antibody to visualize Z-discs. (**A**, **B**) *sls*^*1*^ and *sls*^*j1D7*^ heterozygotes show predominantly normal sarcomere structure. (**C**, **D**) When *sls* mutant alleles are combined with *Mef2>cher-JF* a complete loss of sarcomere structure is observed. Scale bars: 5 μm. (**E**) Quantification of recognizable sarcomeres in different *sls* heterozygotes alone or in combination with *Mef2>cher-JF*. (**F**) *Mef2>cherJF*-associated lethality is also aggravated by addition of *sls* mutant alleles. Statistical significance assessed by one-way ANOVA with post hoc Tukey: * = P ≤ 0.05, ** = P ≤ 0.01, *** = P ≤ 0.001, **** = P ≤ 0.0001. (**G**-**M**) Wing beat frequency recordings in different *cher* and *sls* heterozygotes. The average of 10 flies with standard deviation is shown. (**G**, **H**, **I**) Heterozygous *cher* mutants can fly. (**J**) *sls*^*j1D7*^ heterozygotes are also able to sustain flight. (**K**, **L**) However, *cher*^*Q1415sd*^ *sls*^*j1D7*^ *and cher*^*Δ5*^ *sls*^*j1D7*^ transheterozygotes are not capable of sustained flight. (**M**) *cher*^*Q1042x*^ *sls*^*j1D7*^ transheterozygotes can fly.

To extend our genetic interaction analysis we tested other *sls* alleles. First, we asked whether removal of one copy of *sls* would affect the *Mef2>cher*^*JF02077*^ phenotype. To do so we first analyzed the IFM from *sls*^*1*^ and *sls*^*j1D7*^ heterozygotes. Both *sls*^*1*^ and *sls*^*j1D7*^ appear normal and can fly (Fig 7A and 7B). Yet, when these alleles were combined with *Mef2>cher*^*JF02077*^, most myofibrils fray and sarcomeres disappear (Fig 7C and 7D). This genetic interaction is highly significant for both *sls*^*1*^ and *sls*^*j1D7*^ (Fig 7E). We also measured the lethality in *Mef2>cher*^*JF02077*^ alone or in combination with different *sls* alleles. Consistently, all *sls* alleles significantly enhance the lethality associated with depletion of Cher (Fig 7F). Lastly, we tried a subtler approach, measuring the interaction using our infrared laser tachometer in transheterozygous flies. The *cher* mutant heterozygotes as well as *sls*^*j1D7*^ heterozygotes can sustain flight for more than 30 seconds (Fig 7G–7J). Yet, in *sls*^*j1D7*^ *cher*^*Δ5*^ and *sls*^*j1D7*^*cher*^*Q1415sd*^ transheterozygotes flying ability was severely affected, with flies unable to sustain flight for more than a couple of seconds (Fig 7K and 7L). Surprisingly, *sls*^*j1D7*^ *cher*^*Q1042x*^ could normally sustain flight (Fig 7M). Heterozygotes and transheterozygotes showed no obvious sarcomere defects, when analyzed by confocal microscopy (S4 Fig). Thus, *cher* and *sls* strongly genetically interact across multiple allelic combinations and enhance different *cher* phenotypes, suggesting that the interaction of titin and filamin is of crucial importance to sarcomere structure.

### The C-term Cher Ig domains bind the Sls I-band region

**Fig 8:**
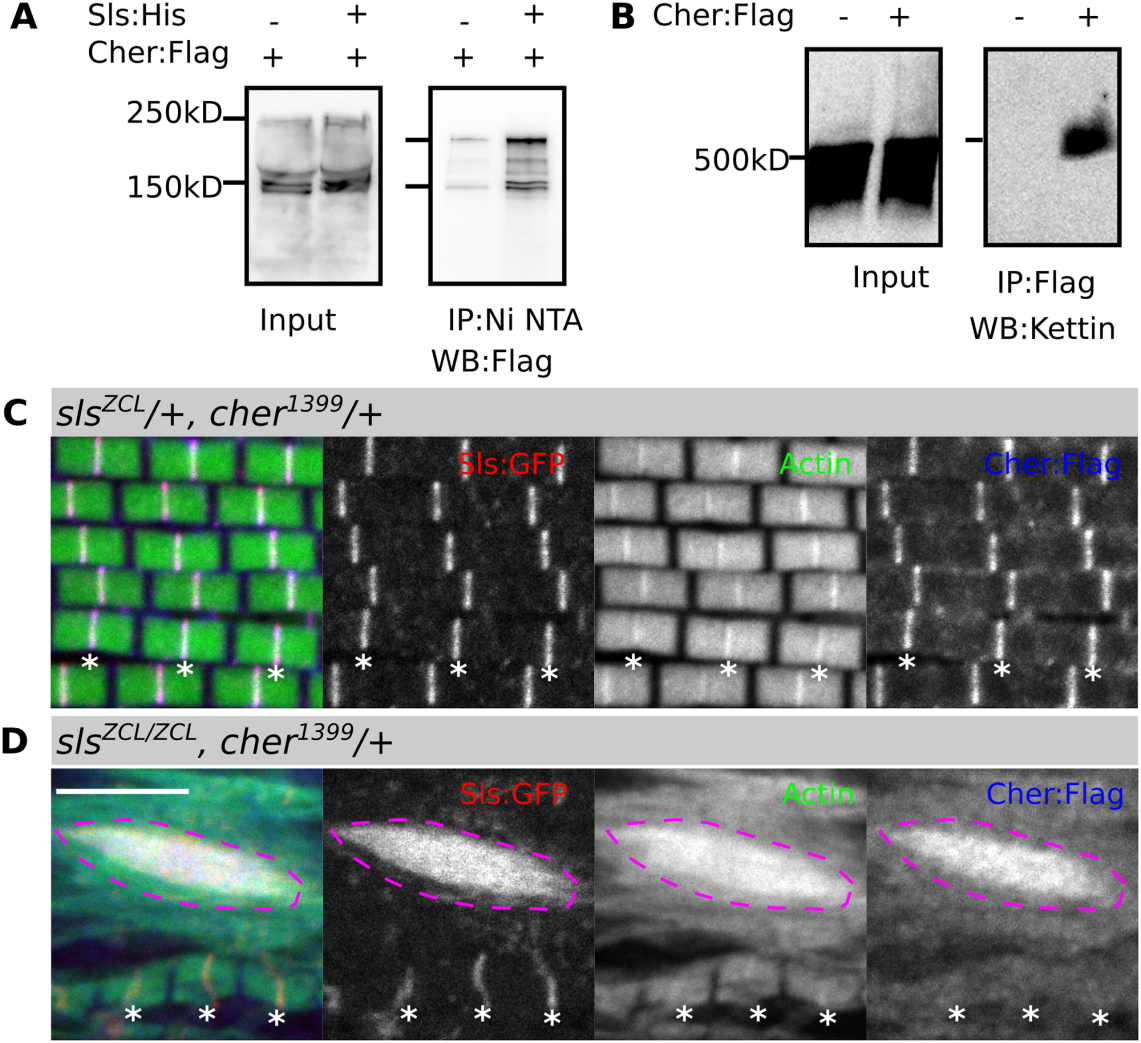
The Sls isoform Kettin binds Cher and recruits Cher to the Z-disc. (**A**) Pull-down of His-tagged Sls co-immunoprecipitates Cher-Flag, in contrast to pull-down with wild type thorax extract. (**B**) Pull-down of Cher-Flag co-immunoprecipitates the Sls isoform Kettin, whereas wild type thorax extract does not. (**C**, **D**) Confocal images of IFM stained with phalloidin to visualize actin thin filaments and anti-Flag antibody to visualize Cher. GFP fluorescence marks Sls at the Z-disc. (**C**) In *sls*^*ZCL2144*^ heterozygotes Sls-GFP and Cher-Flag colocalize at the Z-disc. (**D**) In *sls*^*ZCL2144*^ homozygotes Cher no longer localizes at the Z-disc and is instead recruited to Sls-GFP aggregates (magenta dotted line). Scale bar: 5 μm.

As Cher and Sls/Kettin colocalize at the Z-disc in the IFM and our genetic interaction data indicate they function jointly, we decided to assess the physical interaction of these proteins. As a starting point, we used the *cher*^*CPTI1399*^ (Flag-Venus tag) allele in combination with *sls*^*ZCL2144*^ (His/EGFP-tag), which allows us to immunoprecipitate Sls as bait protein using Ni-beads and detect all interacting Cher isoforms using Flag antibody. In this approach, all Cher isoforms were enriched in Sls-containing beads, compared to the control beads (Fig 8A). This suggested that all Cher isoforms are in a complex with Sls. To test if Kettin, the most common Sls isoform in the IFM, is responsible for Cher binding, we immunoprecipitated *cher*^*CPTI1399*^ with anti-Flag affinity beads and tested for Kettin presence. Again, Kettin was enriched in the Cher-containing beads, suggesting that Cher can bind Kettin in the IFM (Fig 8B).

To independently confirm this interaction, we analyzed the localization of filamin in a titin mutant. In *sls*^*ZCL2144*^ heterozygotes bearing a Flag-tagged allele of Cher (*cher*^*1399*^*/+*) sarcomeres appear normal with both Sls:GFP and Cher:Flag colocalizing at the Z-disc (Fig 8C). However, in *sls*^*ZCL2144*^ homozygotes Sls is reduced at the Z-disc and accumulates into large ectopic protein aggregates (Fig 8D). Intriguingly, Cher-Flag is no longer observed at the Z-disc, but is instead recruited to the large ectopic Sls aggregates (Fig 8D). These results support the notion that Sls binds and recruits Cher into the Z-disc.

We then wondered if we could further narrow our protein-protein binding analysis. Both Sls and Cher are huge proteins not amenable to standard protein purification protocols. However, recent advances in computational approaches to study protein-protein interactions have shown that evolutionarily persistent protein complexes tend to leave a covariation signature [39]. Since protein complexes can be tightly bound, mutations in one component may be compensated with mutations in other components. This evolutionary covariation signature can be thought of as the correlated changes that appear in the coding sequence of two proteins [39–42]. Furthermore, the strength of the correlation tends to be higher at contact areas [43].

**Fig 9:**
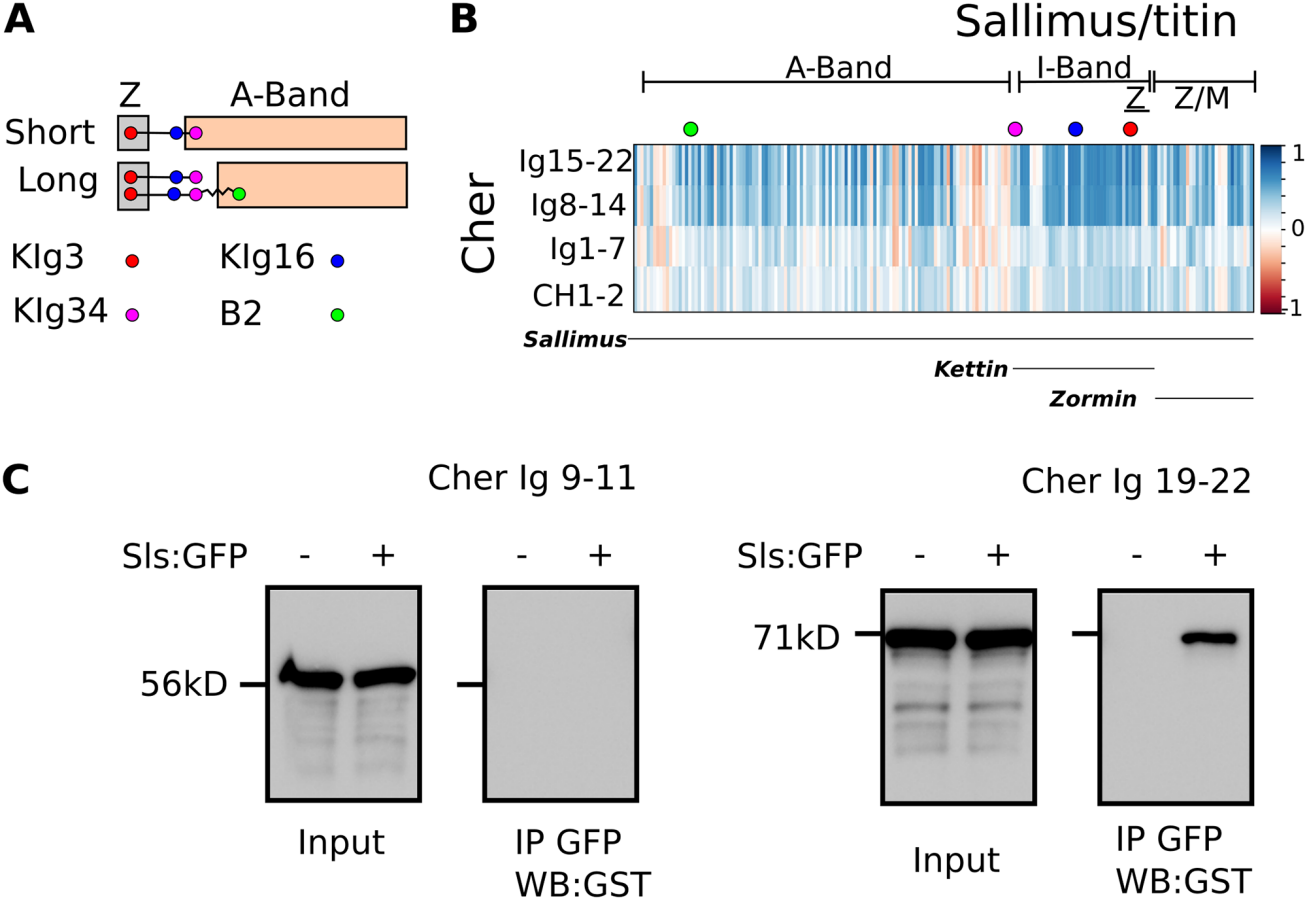
C-terminal Ig domains 19–22 of Cher bind to the Sls isoform Kettin. (**A**) Cartoon showing 4 epitopes in Sls whose precise localization in the I-band has been determined in IFM (short I-band sarcomeres) and leg muscles (long I-band sarcomeres). (**B**) Coevolutionary rate variation analysis of Cher and Sls coding regions. Coevolution scores (-1 to 1) are color-coded, values close to 1 indicate positive coevolution and appear as dark blue. Filamin alignment blocks are shown on the y axis and are labeled, Sls alignment blocks are shown on the x axis and are unlabeled. A large area of dark blue values is observed in the Sls area corresponding to Kettin and Cher C-terminal Ig domains 8–14, and even more pronounced with Ig 15–22. To visualize Sls domains, the position of known epitopes is indicated in colored circles together with their location within A-band, I-band, and Z-disc above the graph. The extent of known Sls isoforms is indicated below the graph. Zormin localizes to Z-disc and M-line. (**C**) Pull-down assay using fly-purified Sls and bacterially expressed Ig domains of Cher. Cher Ig 19–22, but not Cher Ig 9–11 interacts with Sls.

Therefore, we relied on finding the peak of positive evolutionary covariation between *cher* and *sls* as a proxy for their potential contact sites (see Materials and methods). We first divided the coding regions of both genes in alignment blocks and then used those blocks to obtain partial covariation signatures. We divided *cher* and *sls* in 4 and 250 blocks respectively and calculated the covariation between all the possible combinations. A highly-correlated area was found, comprising 2 *cher* blocks, encoding Ig 8–14 and Ig 15–22 and the N-terminus of Sls corresponding to the Kettin isoform (Fig 9A and 9B). These results suggest the C-terminal half of Cher binds to Kettin.

Next, we wanted to know which of the two identified Cher areas mediates the interaction with Sls/Kettin. We therefore expressed and purified two regions of Cher matching the highly coevolving blocks, Cher Ig 9–11 and Cher Ig 19–22 and used them to test for binding against Sls protein purified from thorax extracts. Cher Ig 19–22, but not Cher Ig 9–11 interacts with Sls-GFP (Fig 9C). Thus, the computational results agree with the biochemical data, indicating an interaction of Kettin with the C-terminal Ig 19–22 domains of Cher.

## Discussion

The organization of actin thin filaments is central to the function of the sarcomere and therefore of muscles. The *Drosophila* IFM provides great insight to our current understanding of sarcomere protein assembly and function. Here we investigated the function of Cher, the *Drosophila* filamin homolog.

Filamin was the first actin filament crosslinking protein identified in nonmuscle cells [4]. Filamins act by crosslinking and stabilizing a meshwork of actin filaments [8]. To date, 17 FLNc myopathy-causing variants distributed all along FLNc have been found [44]. The phenotypes of these mutations fall in several different classes, and it is often unclear how the phenotype is caused at a cell biological level [29,45]. Recently, a FLNc mutant in zebrafish was generated revealing a mild muscle fiber phenotype [46], confirming the role of filamin in vertebrate muscles.

Even though human muscles express only one FLNc isoform of around 290 kD, most Cher isoforms are present in the IFM as shown by using different protein trap lines. Like FLNc in vertebrates all Cher isoforms localize to the Z-disc [46,47]. This can now be explained by the titin interaction domain, which we mapped to Cher Ig 19–22, and which is present in all isoforms. Finally, we reveal that Cher depletion leads to three distinct phenotypes: 1) a widened Z-disc, 2) a smaller or fractured Z-disc, and 3) actin incorporation into the H-zone. Consistent with filamin’s known role as an actin-binding protein, these phenotypes are directly linked to the positioning of actin thin filaments.

Distinct phenotypes can be observed in *cher* mutants affecting different isoforms and the most severe phenotype can be obtained by removing all isoforms using RNAi. We propose that the phenotypic differences are due to Cher truncations retaining partial functions. The best example is the *cher*^*Q1042x*^ mutant that creates a truncated form consisting of ABD plus the first 11 Ig domains and leaving the small CherA/B isoforms unaffected: it splits Cher in two halves. Interestingly, *cher*^*Q1042x*^ mutants present a weaker phenotype than *cher*^*Δ5*^ and *cher*^*Q1415sd*^. Retaining function in truncated filamins is not unknown. Previous studies have reported that a truncated Cher consisting of the ABD plus the first 6 Ig domains, but without the dimerization domain, is able to rescue most filamin functions in egg chamber formation [19]. We took advantage of these hypomorphic truncations to analyze in detail the separate functions of the N-terminal ABD and the C-teminal Ig domain regions.

**Fig 10:**
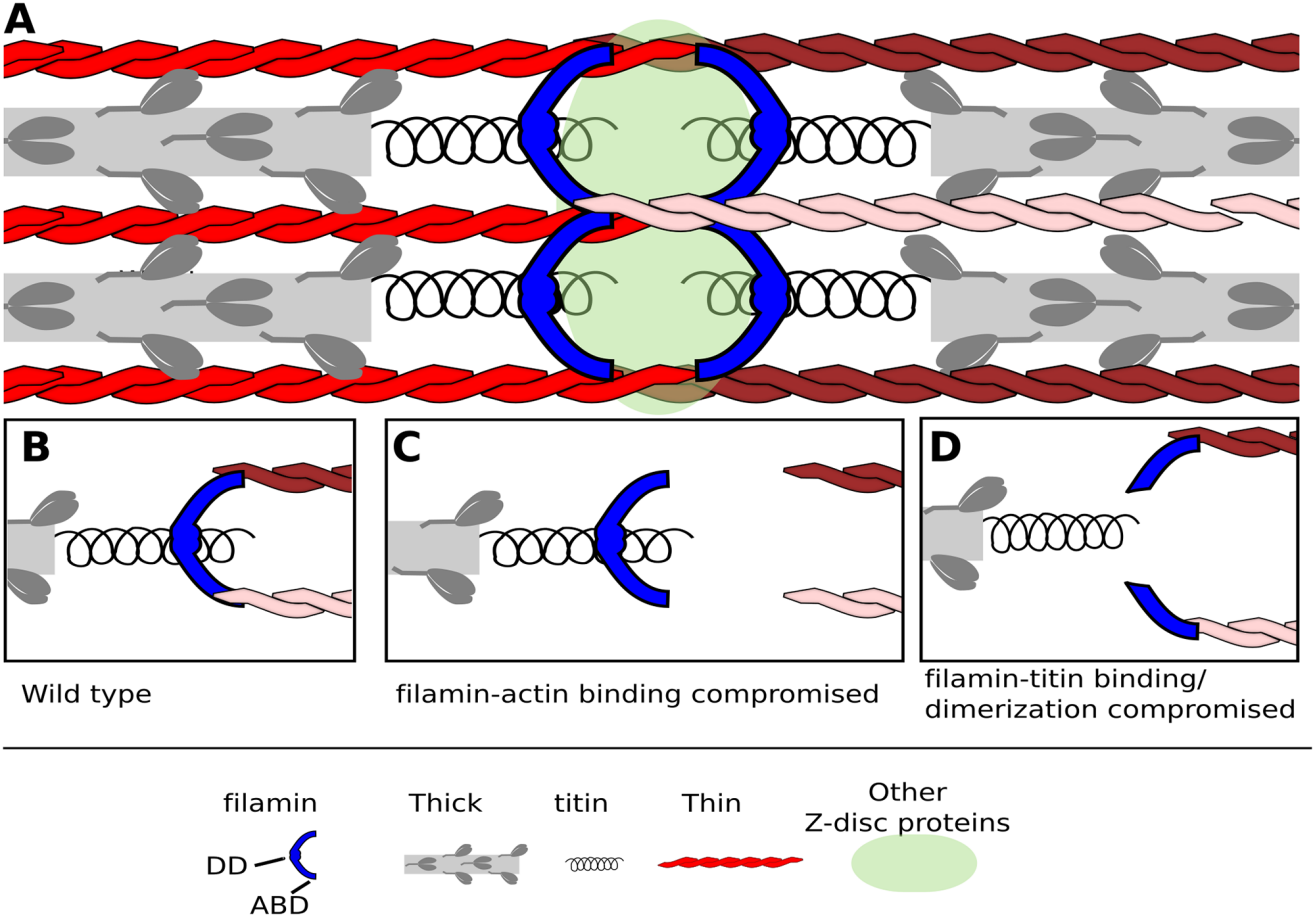
Model of Cher/Filamin function in stabilizing the Z-disc. (A) Filamin dimers (blue) localize to the Z-disc or Z-disc edge, where they serve to stabilize the Z-disc in two directions. A filamin domain close to or identical to the dimerization domain binds titin in one sarcomere, while the two CH domains bind actin thin filaments from the adjacent sarcomere. Thin filaments are in different shades of red in the right half sarcomere to indicate their location in different planes. This configuration allows Cher to stabilize the Z-disc in two directions: parallel and perpendicular to the myofibril. (B-D) Models showing one filamin dimer binding two thin filaments and titin. (B) Wild type. (C) In *cher* mutants that disrupt actin binding, perpendicular stability is partially maintained but anchorage to the Z-disc is lost. Thus, an actin-free and widened Z-disc is often observed. (D) In mutants which compromise Sls binding and dimerization, the Z-disc ruptures perpendicular to the myofibril, causing Z-disc material and thin filaments to detach. In all mutants except *cher*^*Q1042x*^, individual thin filaments can invade the H-zone owing to myosin power strokes and improper anchorage of thin filaments at the Z-disc.

We propose that filamin/Cher is located at the Z-disc where it binds actin thin filaments through the N-terminal ABD and titin/Kettin through its C-terminal Ig domains. Cher is present as a homodimer held together by its most C-terminal Ig domain. This configuration allows filamin to bridge titin/Kettin with actin thin filaments from opposing sarcomeres, maintaining Z-disc cohesion both in parallel and perpendicularly to the sarcomere (Fig 10A and 10B).

### Filamin-titin binding

The giant elastic protein titin is a 1 μm long flexible filament that spans half the sarcomere. In *Drosophila* the titin homolog Sls spans from the Z-disc to the edge of the A-band, providing elasticity between the thick filaments and the Z-disc, as a molecular spring [37,48]. Elasticity comes initially from the extension of the PEVK region, with the lowest mechanical stability, followed by the N2B region and finally the unfolding of the Ig and Fibronectin domains [49,50].

Titin is believed to function as a massive protein scaffold. Consistently, at least 24 direct human titin ligands have been found, with 8 of them in the Z-disc/I-band region including actin, ɑ-actinin, nebulin and FLNc [51]. Filamin is likewise a large scaffold protein. We show that the link previously described biochemically between filamin and titin is conserved in invertebrate muscles and required for the stability of the Z-disc.

First, using four different assays we show that *cher* and *sls* display a strong genetic interaction, suggesting Sls and Cher have a common function in muscles. We then tested for a physical interaction. His-tagged Sls can precipitate all Cher isoforms and inversely, Flag-tagged Cher co-immunoprecipitates Kettin, the most common IFM isoform of Sls. Common to all Cher isoforms is the last C-terminal region containing the last 8 Ig domains. Experiments with bacterially purified Cher Ig domains demonstrate the requirement of the last four Ig domains of Cher for this interaction. Thus, Sls-Cher binding happens near the dimerization domain of Cher. A similar *in vitro* binding has been shown between the Zis1-2 region of human titin to the Ig 20–24 domains of both FLNa and FLNc [52]. While we cannot rule out an indirect binding, because Sls could only be purified from thorax extracts, our results add that filamin-titin binding is crucial for sarcomere stability and indicates that this interaction is conserved.

Lastly, we provide direct evidence for the function of Cher-Sls binding. Removing the last C-terminal 8 Ig domains of Cher with the *cher*^*Q1415sd*^ mutant, thereby removing the Sls-binding site, results in a smaller and fractured Z-disc, and actin incorporation into the H-zone. A smaller Z-disc is a very representative phenotype. Apart from *cher*, it has only been reported for *sls* [38].

In summary, Cher binds the I-band region of Sls/Kettin via C-terminal Ig domains; *cher* and *sls* genetically interact; and removal of the Sls/Kettin-binding region in Cher leads to a *sls*-like phenotype resulting in Z-discs that are smaller or fractured along the perpendicular axis (Fig 10D). Our results therefore strongly suggest that filamin and titin are part of a complex mediating Z-disc stability.

### Filamin-actin binding

Maintaining actin thin filaments aligned and anchored is the central function of the Z-disc protein complex. Thus, many Z-disc proteins directly bind actin. Not surprisingly, filamin, a well-studied actin-binding protein localizes at the Z-disc where it binds actin through a conserved N-terminal ABD, composed of two CH domains.

We now show that filamin-actin association is critical for Z-disc cohesion, by showing a widened Z-disc phenotype for different *cher* mutants that specifically affect the isoform containing the ABD or by specifically disrupting the ABD. Further, we show a genetic interaction between these *cher* alleles and *Act88F*, confirming a functional filamin-actin link in muscles.

The widened Z-disc phenotype is unique compared to other IFM phenotypes described, in agreement with the proposed specific filamin-actin function in the Z-disc. The Z-disc widens and an actin-free area appears in place of the Z-disc (Fig 10C). However, the adjacent sarcomeres are still well organized and the phenotype is also unevenly distributed across myofibrils. This suggests that filamin is not required for the initial assembly of myofibrils, but rather for maintaining sarcomere structure during repetitive contractile load. In line with this notion, the Z-disc widens parallel to the vector of sarcomere contraction. A somewhat similar widened Z-disc phenotype is seen upon stretching isolated myofibrils using a piezoelectric micromotor [37]. Further, a filamin mutation in medaka, which leads to myofibril degeneration, can be rescued by inhibiting muscle contraction [53], and filamin has been shown to localize to sarcomeric microlesions formed upon strong contraction and mediate repair [54]. This again supports the proposal that stretching forces caused by muscle contractions produce the Z-disc widening phenotype in CherD mutants. This phenotype is specific to mutations of the filamin ABD, while maintaining Z-disc cohesion perpendicular to the sarcomere is likely mediated by both filamin-actin and filamin-titin binding.

Z-discs with higher levels of actin at the Z-disc were sometimes seen upon disruption of Cher ABD-containing isoforms. One explanation for this could be the better accessibility of epitopes for binding of phalloidin in the widened Z-disc sarcomeres. It is well documented that antibodies cannot penetrate IFM myofibrils well, e.g. the myosin antibody stains only at the A/I-junction and the M-line [55]. Tearing and widening of Z-discs may occasionally expose additional epitopes on thin filaments leading to the appearance of higher levels of actin.

Finally, we show that both mutating the ABD in *cher*^*Δ5*^ or the Sls-binding/dimerization domains in *cher*^*Q1415sd*^ leads to actin filaments invading the H-zone. We propose that in both cases individual thin filaments are no longer stably anchored at the Z-disc, and can therefore be occasionally moved by myosin power strokes into the H-zone. Cher provides thin filaments with the necessary anchorage and elastic support to remain attached to the Z-disc. Finally, as both *cher*^*Δ5*^ and *cher*^*Q1415sd*^ mutants share this phenotype and because the addition of a mutated copy of *sls* greatly enhances this phenotype, we propose that both titin-binding and actin-binding are required for keeping thin filaments anchored (Fig 10C and 10D).

Importantly, we show a direct structural role for filamin in addition to the signalling and mechanosensing role ascribed to filamin in muscles so far [7]. We have shown for the first time that filamin crosslinks parallel actin filaments with the widened Z-disc phenotype, in addition to the previously demonstrated perpendicular crosslinking in nonmuscle cells [9]. Related to the Z-disc structure, our data show that Z-discs require much more actin crosslinking than just by ɑ-actinin to withstand the strong contractile forces acting on them.

## Materials and methods

### Fly stocks and genetics

Unless specified, all crosses were done at 25°C. The following fly stocks were obtained from the Bloomington *Drosophila* stock center: *Mef2-Gal4*, *cher*^*CPTI1399*^, *cher*^*CPTI847*^ and *cher*^*CPTI1403*^ [31]; cher^MI07480-GFSTF.0^, an insertion that integrates a Flag and an EGFP tag [32]; the RNAi transgene *cher*^*JF02077*^; the *sls*^*ZCL2144*^ allele (Sls-GFP) is a protein trap that incorporates a His-Tag and a EGFP sequence into all annotated *sls* transcripts [38]; *sls*^*1*^ (*D-Titin*^*14*^) is an EMS amorph mutant and *sls*^*j1D7*^ is a *P{lacW}* insertion into an exon encoding part of the PEVK-2 domain that fails to complement *sls*^*1*^ and *sls*-uncovering deficiencies [56]; the deficiency line *Df(3R)Exel6176* uncovers the entire *cher* locus and was used in combination with all *cher* alleles. The RNAi transgene *cher*^*KK107451*^ was obtained from the Vienna *Drosophila* RNAi Center. The *cher*^*Δ5*^ mutant is a deletion that removes the CherD transcription start site, was made by imprecise excision of a P-element; *cher*^*1*^, *cher*^*Q1415sd*^ and *cher*^*Q1042X*^ are EMS mutants; *cher*^*Q1415sd*^ and *cher*^*Q1042X*^ introduce a stop codon in positions 1415 and 1042, respectively; all were a kind gift from Lynn Cooley. Cher Protein Trap lines *cher*^*CPTI1399*^, *cher*^*CPTI847*^ and *cher*^*CPTI1403*^ were obtained from the *Drosophila* Genomics and Genetic Resources at Kyoto Institute of Technology [31]. The *UAS-CherA-GFP* stock was a kind gift from Sven Huelsmann [27].

### IFM dissection and staining

IFM dissection was done as previously described [57, 58]. Half thoraces were glycerinated (20 mM Na-Phosphate pH 7.2, 2 mM MgCl_2_, 2 mM EGTA, 5 mM DTT, 0.5% Triton X-100, 50% glycerol) overnight at -20°C. IFMs were dissected, washed and then fixed with 4% paraformaldehyde in relaxing solution (20 mM Na-Phosphate pH 7.2, 2 mM MgCl_2_, 2 mM EGTA, 5 mM DTT, 5 mM ATP) with protease inhibitors (Roche). The following primary antibodies were used: rat anti-Actinin MAC276 (1:100, Babraham Bioscience Technologies), mouse anti-Flag 1:400 (Sigma-Aldrich), rat anti-Kettin KIg16 MAC155 (1:400, Babraham Bioscience Technologies). Primary antibody incubation was carried out overnight in PBS-0.1% Triton X-100 and secondary antibodies of the Alexa series (ThermoFisher Scientific) used at a 1:400 dilution and TRITC-phalloidin were incubated in PBS for 2 hours. Samples were mounted in ProLong Gold antifade solution (ThermoFisher Scientific).

### Confocal microscopy

All images were acquired using a 63x 1.4 NA HC Plan Apochromat oil objective on a Leica SP8 confocal microscope. Properly stained muscles were manually selected and aligned so that fibers run left to right. Once a muscle fiber was selected and aligned, random areas were imaged at 9x further magnification corresponding to 420 μm^2^ at an image resolution of 1024 x 1024 pixels. All quantifications were done at the same magnification and resolution to assure homogeneity.

We used 488 nm-20 mW, 552 nm-20 mW, and 638 nm-30 mW lasers. Emitted light was detected with PMT and HyD detectors. Laser power was typically set between 1% and 3%, the pinhole was set to 1 airy unit and the gain was set between 700 V and 900 V (PMT) or between 10 V and 100 V (HyD). Smart offset was kept at 0%. Scanning speed was set to 400 Hz. Comparable settings were used for all image acquisitions.

### Image quantifications

Missing sarcomeres were estimated as a decrease in the total number of distinguishable sarcomeres per image.

Actin accumulation was obtained by measuring phalloidin-TRITC staining grayscale values in the H-zone divided by the same measurement in the zone between the H-zone and the I-band, where actin is normally present. To ensure homogeneity all measurements were done using a 0.9 x 0.9 μm region of interest.

Cher intensity at the Z-disc was measured using the ImageJ plot profile tool line, which displays the intensities of pixels along a line. The X-axis represents distance (μm). To better compare pixel intensities from different flies, the pixel intensities were first normalized for each individual image. Average profiles for each Cher trap line were computed from 10 images and plotted with RStudio.

### Lethality quantifications

Embryos bearing the correct genotype were selected for the absence of GFP fluorescence (*TM3*, *twist-Gal4*, *UAS-GFP*, *Sb*) and incubated at a controlled temperature. The resulting adults were counted and the lethality ratio was calculated.

### Statistical analysis

Statistical significance in all figures was assessed using one-way ANOVA followed by post-hoc Tukey tests (GraphPad Prism 7) and plotted as a box plot with notches representing 95% confidence intervals using the boxplot{graphics} function in R software.

### Immunoprecipitation and immunoblotting

50 adult fly thoraces were homogenized in lysis buffer (20 mM Tris- HCl pH 8, 100 mM NaCl, 1 mM MgCl_2_, 1 mM DTT, 5% glycerol, 0.5% Triton X-100 and complete EDTA-free protease inhibitor; Roche). Protein extracts were then incubated with prewashed anti-FLAG M2 affinity resin (Sigma-Aldrich) or Ni NTA agarose beads for 3 hours at 4°C. After incubation, the beads were washed twice with wash buffer (20 mM Tris-HCl pH 8, 150 mM NaCl, 5% glycerol, 0.2% Triton X-100). Bound proteins were eluted by boiling in 2x SDS sample buffer. Eluates were analyzed by SDS-PAGE and by immunoblotting.

For Sls-GFP purification, 100 adult fly thoraces were homogenized in lysis buffer. Protein extracts were then incubated with prewashed anti-GFP magnetic beads (ChromoTek) for 3 hours at 4°C. After incubation, the beads were washed twice with wash buffer (20 mM Tris-HCl pH 8, 250 mM NaCl, 5% glycerol, 0.2% Triton X-100). Sls-containing beads were then incubated with bacterially expressed proteins. Following incubation, the beads were again washed twice with wash buffer and bound proteins were eluted by boiling in 2x SDS sample buffer.

Cher Ig 19–22 and Cher Ig 9–11 were cloned into the pGEX-5X-1 vector between EcoRI and XhoI for bacterial expression. *E*. *coli* strain BL-21 bacteria expressing GST-tagged recombinant proteins were lysed by sonication in 20 mM Tris-HCl pH 8, 200 mM NaCl, 1 mM MgCl_2_, 1 mM DTT, 5% glycerol, 0.2% Triton X-100, 1 mg/ml lysozyme and complete EDTA-free protease inhibitor (Roche).

Immunoblotting antibodies were used at the following dilution: rat anti-Kettin KIg16 MAC155 (1:4000, Babraham Bioscience Technologies); mouse anti-FLAG antibody at 1:5000 (Sigma-Aldrich). The immunoreaction was visualized by ECL (Millipore).

### Homology modeling

CherD protein structure was calculated through the RaptorX protein structure prediction server using Cher-PA as query and FLNA, B or C as templates. The best resulting structure was analyzed and colored using Chimera UCSC software. To generate CherD^847^ and CherD^1403^ structures, a predicted protein sequence based on Cher-PA and Venus-GFP was generated according to the protein trap insertion site. CherD^847^ introduces a Venus trap in between Ig 11 and Ig 12, while in CherD^1403^, the Venus trap is inserted into the first CH domain. The resulting sequences were modelled using RaptorX [59].

### Evolutionary rate covariation

Protein covariation was calculated as previously described [40–42]. Briefly, exon-coding regions for each gene were obtained through the Table Browser at the UCSC genome browser (genome.ucsc.edu). BED files containing Augustus exon predictions for each gene from the R5/dm3 genome assembly were sent to the Galaxy website (www.usegalaxy.org). Alignments from 12 *Drosophila* species were directly obtained from Galaxy-stored MultiZ alignments in MAF format. MAF files were converted to Fasta format and imported to R software using read.dna{ape}. Pairwise distances were calculated for each species pair using dist.dna{ape} and transformed into their relative distances [60]. Finally, the Pearson correlation coefficient was used to compare relative distances using cor{stats} and plotted using corrplot{corrplot}.

### Wing beat frequency assay

The wing beat frequency of a fly was determined using an optical tachometer as previously described [58]. In this study, 5-7-day old flies were first glued on pipette tips, followed by measurement of wing beat frequency for 5 minutes using an infrared laser tachometer (Model UT372, Uni-Trend Technology). A 30-second continuous flight window was selected from the 5-minute flight record.

### Electron microscopy

Thoraces were treated with 5 mM MOPS pH 6.8, 150 mM KCl, 5 mM EGTA, 5 mM ATP, 1% Triton X-100 for 2 hours at 4°C, followed by overnight incubation in the same buffer without Triton X-100 but 50% glycerol. Samples were then washed in rigor solution (5 mM MOPS pH 6.8, 40 mM KCl, 5 mM EGTA, 5 mM MgCl_2_, 5 mM NaN_3_) and fixed in 3% glutaraldehyde, 0.2% tannic acid in 20 mM MOPS pH 6.8, 5 mM EGTA, 5 mM MgCl_2_, 5 mM NaN_3_ for 2 hours at 4°C. Secondary fixation and embedding were as described before [57]. Images of recognizable sarcomeres were acquired on a Tecnai 12 BioTwin 120 kV transmission electron microscope with an AMT XR80C CCD camera (FEI).

## Supporting information

S1 Fig Cher localization and phenotype. (**A**, **B**) Confocal images of IFM stained with phalloidin to visualize actin thin filaments and stained with anti-Flag antibody to visualize Cher or showing GFP fluorescence to visualize Cher-GFP. (**A**) The *cher*^*CPTI847*^ protein trap localizes to the Z-disc. (**B**) The smallest Cher isoform (*Mef2-Gal4*, *UAS-cherA-GFP*) localizes to the Z-disc. (**C**) Line scan plot of intensity values at the Z-disc of Cher Trap lines stained with anti-Flag antibody showing comparable values for the three lines. **(D)** Immunoblots from thorax extracts of three Cher protein traps, *cher*^*CPTI1399*^, *cher*^*GFSFT*^ and *cher*^*CPTI1403*^ incubated with anti-Flag antibody. (**E, F**) Confocal images of IFM stained with phalloidin to visualize actin thin filaments and with anti-Kettin antibody to visualize Z-discs. (**E**) *cher* RNAi line *cher-KK107451* shows similar phenotypes as *cher-JF02077*, that is, sarcomere disorganization and widened Z-discs. (**F**) Depletion of Cher using both RNAi lines results in a stronger sarcomere phenotype than either RNAi line alone. Scale bars: 10 μm.
(TIF)

S2 Fig Cher mutants and homology modelling of Cher-Venus fusions. (**A**) Schematic representation of *cher* mutants and predicted isoforms. The insertion sites for the four protein trap mutants used are shown as green triangles. The two point mutations (*cher*^*Q1415sd*^ and *cher*^*Q1042x*^) that introduce an early stop codon are shown by a black asterisk. The *cher*^*Δ5*^-deleted segment is depicted as a line. Complete *cher* gene span is shown as a continuous orange line. Selected isoforms are shown in blue, grouped into 4 groups as in Fig 1. In *cher*^*Δ5*^ homozygotes CherD isoforms are lost. In *cher*^*Q1415sd*^ homozygotes all isoforms are truncated, leaving the last 7 Ig domains untranslated. In *cher*^*Q1042x*^ homozygotes CherA/B isoforms are not affected and CherC/D isoforms are truncated after Ig domain 11, resulting in a Cher protein split into two halves. (**B**) CherD (Flybase Cher-PA) monomer homology model based on FLNa and FLNc structures produced by RaptorX protein structure prediction server. Conserved domains obtained from the NCBI database are shown: CH ABD domain (blue), Ig domains (orange) and dimerization domain (magenta). (**C**) Homology model for CherD^847^, which incorporates a Venus-Flag tag (green) in between Ig domains 11 and 12. (**D**) Homology model for CherD^1403^, which incorporates the same Venus-Flag tag in the first CH domain.
(TIF)

S3 Fig *cher* mutant phenotypes. Confocal images of IFM stained with phalloidin to visualize actin thin filaments and anti-ɑ-Actinin antibody to visualize Z-discs. (**A**) Control sarcomeres showing ɑ-Actinin staining at the Z-disc. (**B**) Actin accumulation at the H-zone can also occasionally be seen in *cher*^*Δ5*^ mutants, consistent with TEM results. Scale bars: 10 μm.
(TIF)

S4 Fig IFM from *sls*^*J1D7*^ *cher* transheterozygous mutants. Confocal images of IFM stained with phalloidin to visualize actin thin filaments in green and anti-Kettin antibody to visualize Z-discs in red. Scale bars: 5 μm. All heterozygous or transheterozygous combinations are predominantly wild type in appearance.
(TIF)
