# Supplementary figures and images for: Filamin actin-binding and titin-binding fulfill distinct functions in Z-disc cohesion
Source: PLoS Genet. 2017 Jul 21;13(7):e1006880. doi: 10.1371/journal.pgen.1006880 (PMC5521747; doi:10.1371/journal.pgen.1006880)

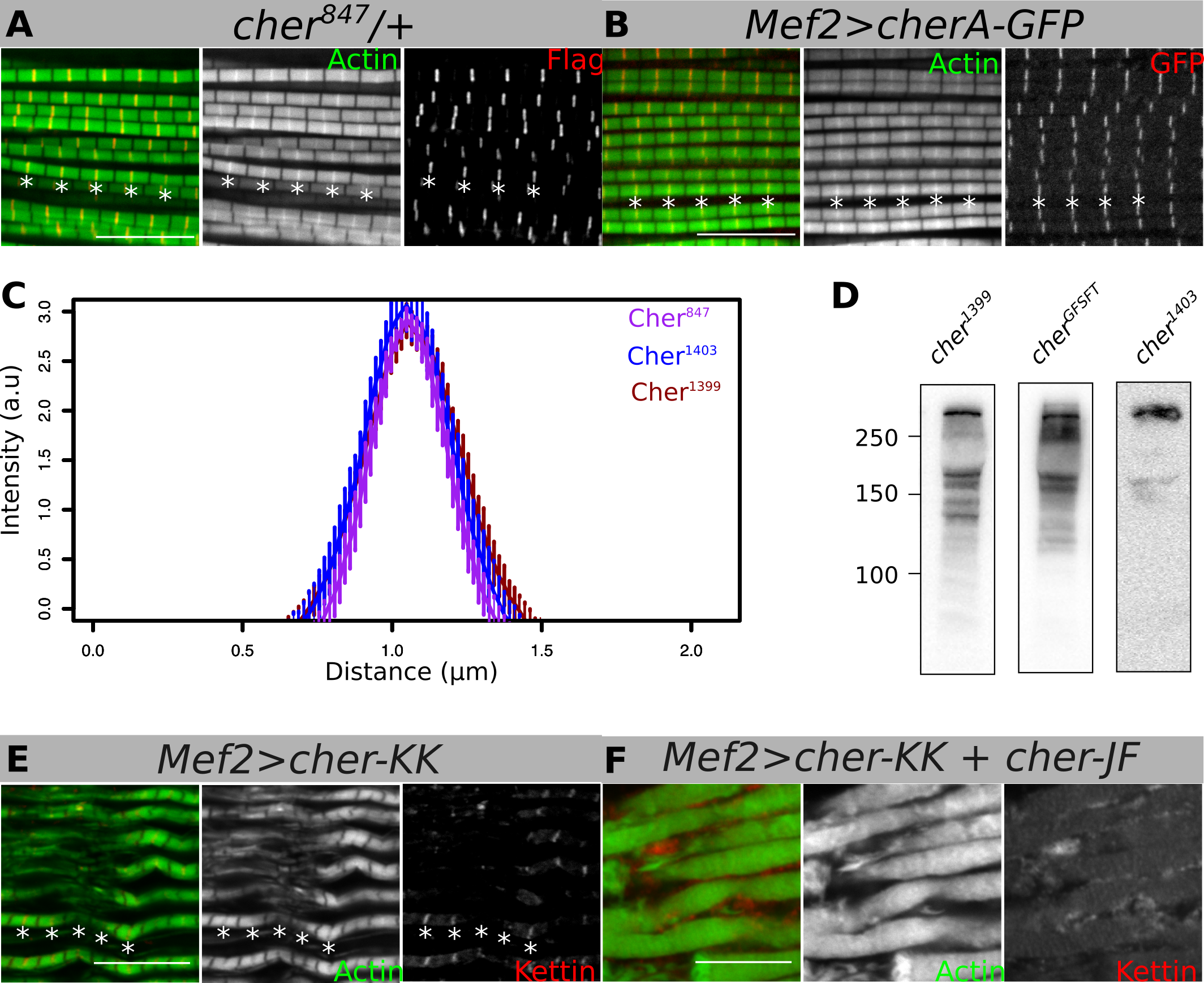

Supplement: S1 Fig — (A, B) Confocal images of IFM stained with phalloidin to visualize actin thin filaments and stained with anti-Flag antibody to visualize Cher or showing GFP fluorescence to visualize Cher-GFP. (A) The cherCPTI847 protein trap localizes to the Z-disc. (B) The smallest Cher isoform (Mef2-Gal4, UAS-cherA-GFP) localizes to the Z-disc. (C) Line scan plot of intensity values at the Z-disc of Cher Trap lines stained with anti-Flag antibody showing comparable values for the three lines. (D) Immunoblots from thorax extracts of three Cher protein traps, cherCPTI1399, cherGFSFT and cherCPTI1403 incubated with anti-Flag antibody. (E, F) Confocal images of IFM stained with phalloidin to visualize actin thin filaments and with anti-Kettin antibody to visualize Z-discs. (E) cher RNAi line cher-KK107451 shows similar phenotypes as cher-JF02077, that is, sarcomere disorganization and widened Z-discs. (F) Depletion of Cher using both RNAi lines results in a stronger sarcomere phenotype than either RNAi line alone. Scale bars: 10 μm. (TIF) [file pgen.1006880.s001.tif]

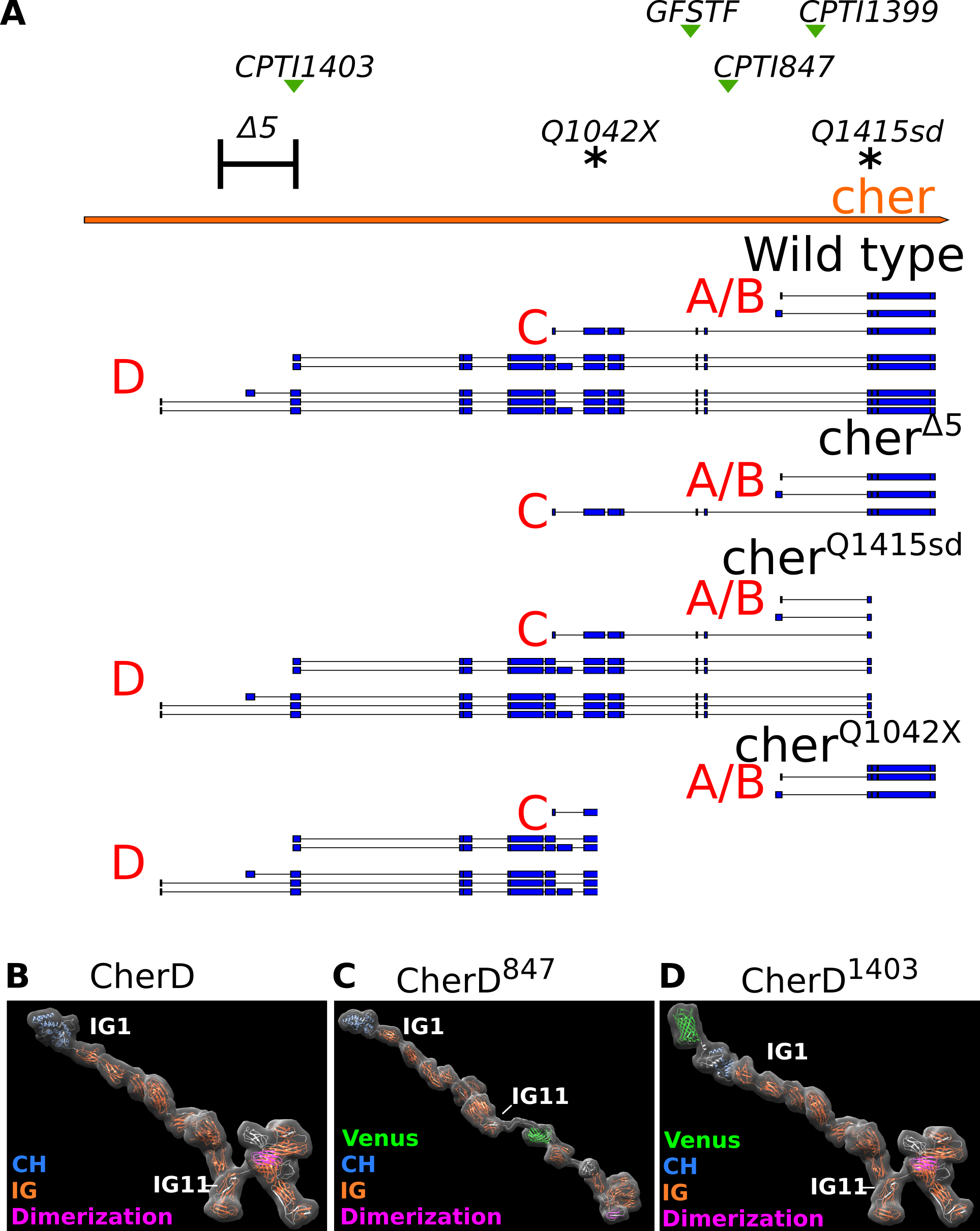

Supplement: S2 Fig — (A) Schematic representation of cher mutants and predicted isoforms. The insertion sites for the four protein trap mutants used are shown as green triangles. The two point mutations (cherQ1415sd and cherQ1042x) that introduce an early stop codon are shown by a black asterisk. The cherΔ5-deleted segment is depicted as a line. Complete cher gene span is shown as a continuous orange line. Selected isoforms are shown in blue, grouped into 4 groups as in Fig 1. In cherΔ5 homozygotes CherD isoforms are lost. In cherQ1415sd homozygotes all isoforms are truncated, leaving the last 7 Ig domains untranslated. In cherQ1042x homozygotes CherA/B isoforms are not affected and CherC/D isoforms are truncated after Ig domain 11, resulting in a Cher protein split into two halves. (B) CherD (Flybase Cher-PA) monomer homology model based on FLNa and FLNc structures produced by RaptorX protein structure prediction server. Conserved domains obtained from the NCBI database are shown: CH ABD domain (blue), Ig domains (orange) and dimerization domain (magenta). (C) Homology model for CherD847, which incorporates a Venus-Flag tag (green) in between Ig domains 11 and 12. (D) Homology model for CherD1403, which incorporates the same Venus-Flag tag in the first CH domain. (TIF) [file pgen.1006880.s002.tif]

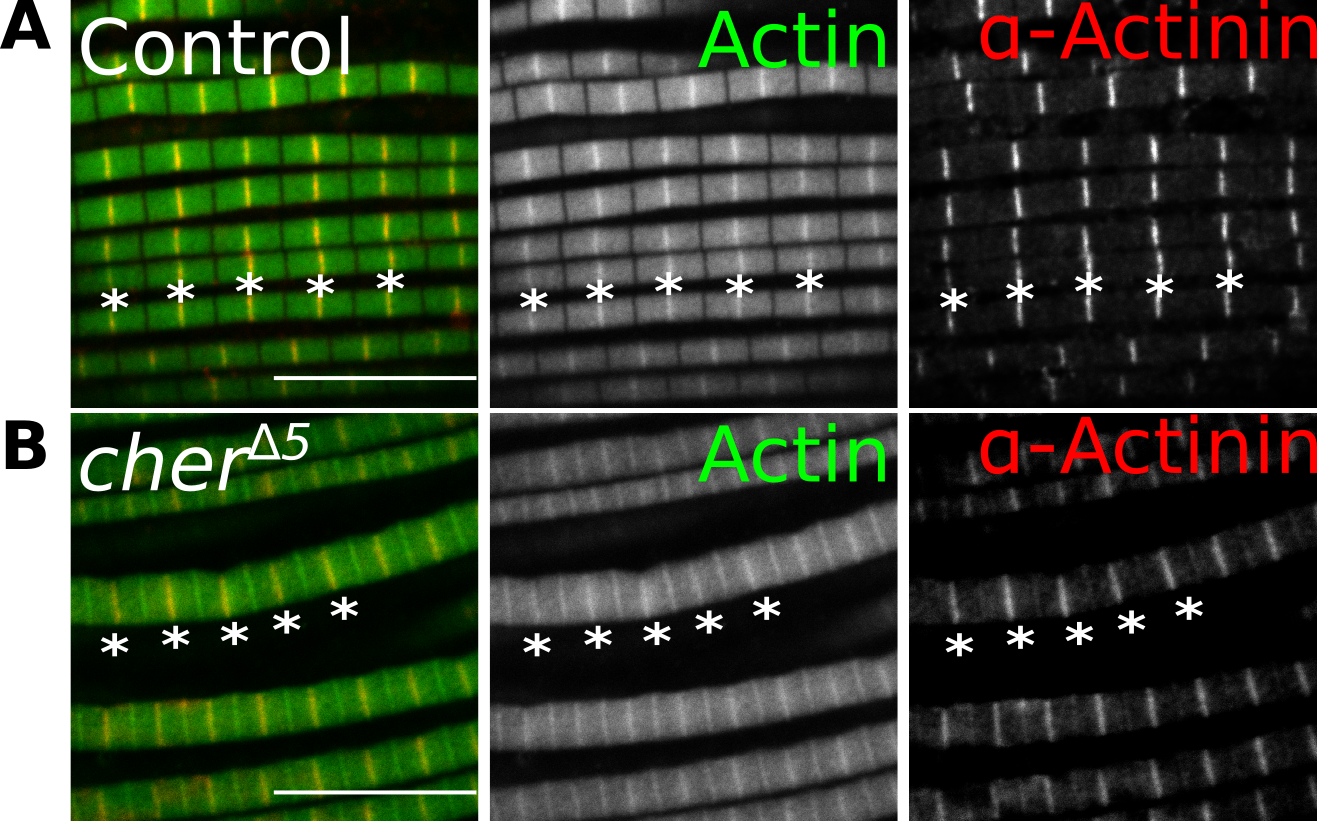

Supplement: S3 Fig — Confocal images of IFM stained with phalloidin to visualize actin thin filaments and anti-ɑ-Actinin antibody to visualize Z-discs. (A) Control sarcomeres showing ɑ-Actinin staining at the Z-disc. (B) Actin accumulation at the H-zone can also occasionally be seen in cherΔ5 mutants, consistent with TEM results. Scale bars: 10 μm. (TIF) [file pgen.1006880.s003.tif]

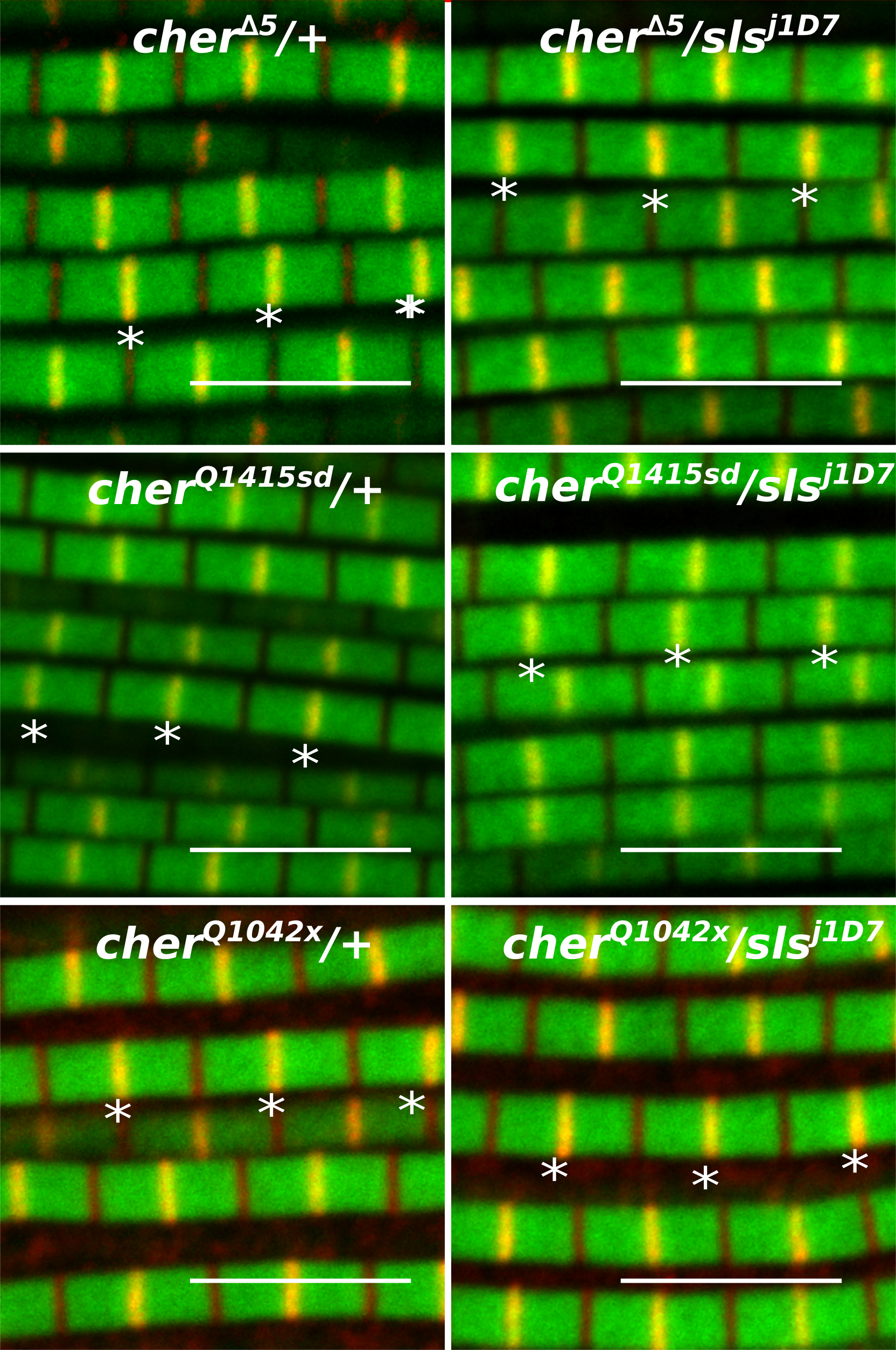

Supplement: S4 Fig — Confocal images of IFM stained with phalloidin to visualize actin thin filaments in green and anti-Kettin antibody to visualize Z-discs in red. Scale bars: 5 μm. All heterozygous or transheterozygous combinations are predominantly wild type in appearance. (TIF) [file pgen.1006880.s004.tif]
